# Population genetic structure and association mapping for iron toxicity tolerance in rice

**DOI:** 10.1371/journal.pone.0246232

**Published:** 2021-03-01

**Authors:** S. Pawar, E. Pandit, I. C. Mohanty, D. Saha, S. K. Pradhan

**Affiliations:** 1 Crop Improvement Division, ICAR-National Rice Research Institute, Cuttack, Odisha, India; 2 Department of Bio-Science and Bio-Technology, Fakir Mohan University, Balasore, Odisha, India; 3 Department of Biotechnology, College of Agriculture, OUAT, Bhubaneswar, Odisha, India; North Dakota State University, UNITED STATES

## Abstract

Iron (Fe) toxicity is a major abiotic stress which severely reduces rice yield in many countries of the world. Genetic variation for this stress tolerance exists in rice germplasms. Mapping of gene(s)/QTL controlling the stress tolerance and transfer of the traits into high yielding rice varieties are essential for improvement against the stress. A panel population of 119 genotypes from 352 germplasm lines was constituted for detecting the candidate gene(s)/QTL through association mapping. STRUCTURE, GenAlEx and Darwin softwares were used to classify the population. The marker-trait association was detected by considering both the Generalized Linear Model (GLM) and Mixed Linear Model (MLM) analyses. Wide genetic variation was observed among the genotypes present in the panel population for the stress tolerance. Linkage disequilibrium was detected in the population for iron toxicity tolerance. The population was categorized into three genetic structure groups. Marker-trait association study considering both the Generalized Linear Model (GLM) and Mixed Linear Model (MLM) showed significant association of leaf browning index (LBI) with markers RM471, RM3, RM590 and RM243. Three novel QTL controlling Fe-toxicity tolerance were detected and designated as *qFeTox4*.*3*, *qFeTox6*.*1* and *qFeTox10*.*1*. A QTL reported earlier in the marker interval of C955-C885 on chromosome 1 is validated using this panel population. The present study showed that QTL controlling Fe-toxicity tolerance to be co-localized with the QTL for Fe-biofortification of rice grain indicating involvement of common pathway for Fe toxicity tolerance and Fe content in rice grain. Fe-toxicity tolerance QTL *qFeTox6*.*1* was co-localized with grain Fe-biofortification QTLs *qFe6*.*1* and *qFe6*.*2* on chromosome 6, whereas *qFeTox10*.*1* was co-localized with *qFe10*.*1* on chromosome 10. The Fe-toxicity tolerance QTL detected from this mapping study will be useful in marker-assisted breeding programs.

## Introduction

Iron is an important micronutrient for rice plant growth and development. It is a constituent of many enzymes of rice plant. Shortage of this element to rice plant reduces growth, development and yield. The element is a co-factor in many enzymes useful in photosynthesis, structural constituent in chlorophyll, proteins, mitochondrial respiration, metal homeostasis and nucleic acid synthesis [1–6]. However, uptake in higher concentration of Fe is toxic to rice plant [7–17]. Toxicity stress is usually seen in lowland rice. Soluble form of this ion (Fe^2+^) is abundant in this ecology. It produces reactive oxygen species (ROS) and hydroxyl radicals (OH) under Fe toxicity condition. These compounds damage rice plants and reduces grain yield drastically. The yield reduction is upto 30% in West and Central Africa [18–20]. Reduction in grain yield due to this stress is high in Burundi, Sierra leone, Burkina Faso, Liberia, Togo, Nigeria, Senegal, Srilanka, Philippines, Malaysia, Indonesia, Vietnam, Thailand, Malaysia, Colombia, Benin, Ivory Coast, Niger, Gambia, Guinea, Guinea-Bissau and Liberia [21–24]. This is also a problem in few locations in Kerala, Coastal and hilly zones of Karnataka, Tamil Nadu, valley soils in Odisha and north eastern region of Meghalaya in India [17, 23, 24]. Existence of genetic variation for Fe-toxicity tolerance in rice is reported in many publications [17, 25–30]. Gene(s)/QTL controlling this stress tolerance and development of robust markers for transfer of tolerance gene into high yielding rice varieties are required for Fe-toxicity tolerance improvement in rice. The genes/QTL controlling tolerance to iron toxicity is complex in nature and governed by many genes. Few quantitative trait loci (QTL) located on different chromosomes have been reported from different mapping populations [25, 28–31]. The involvement of chromosomal regions between 25 to 30 Mb on chromosome 1 and between 0 and 5 Mb on chromosome 3 were reported for tolerance response to the stress [23, 27, 28, 30]. Many publications on transporter genes involved in toxicity tolerance have been well reported [26, 32–37]. However, association of robust markers with major locus controlling this stress tolerance has been rarely reported, validated and used in rice improvement programs. The available results of tolerance genes were reported based on bi-parental mapping populations. Association mapping using large number of genotypes may help for identifying a greater number of loci responsible for Fe-toxicity tolerance in rice.

In this investigation, we shortlisted 119 genotypes and constituted the panel population from the field evaluation of 352 germplasm lines. We phenotyped the panel population under Fe-toxicity field and control conditions for tolerance to iron toxicity. The panel population was genotyped using 51 molecular markers including 47 SSR and 4 gene specific markers covering 12 chromosomes to know the association of markers with Fe toxicity tolerance through marker-trait association in the panel. The detected markers may be useful for Fe-toxicity tolerance breeding programs in rice.

## Materials and method

### Plant material, experimental site and design

A total of 352 rice germplasm lines were evaluated under Fe-toxicity field at Orissa University of Agriculture and Technology (OUAT), Bhubaneswar to constitute a panel population for association mapping study. The germplasm lines consisted of landraces and released cultivars maintained at ICAR-National Rice Research Institute (NRRI), Cuttack and OUAT, Bhubaneswar were used for the investigation (S1 Table). The genotypes were grown in the Fe-toxicity plot in an augmented block design keeping 7 blocks and allotting 52 genotypes to each block including two check varieties during wet season, 2016. The landrace, Dhusura and variety Sebati were taken as tolerant and susceptible checks, respectively in the screening experiment. A panel containing representative population from 352 genotypes was prepared for genotyping and further phenotyping purposes. The genotypes were shortlisted from all the phenotypic groups based on tolerance response to Fe-toxicity (Table 1). The panel population was phenotyped for Fe toxicity stress tolerance in the field during wet seasons, 2017 and 2018. The phenotyping for leaf bronzing index under controlled condition was performed during dry season, 2018.

**Table 1:** Mean leaf bronzing, Fe-content, grain yield and component traits in the shortlisted genotypes under Fe-toxicity stress during wet season, 2017 and 2018.

Sl. No.	Genotypes	Traits under Fe-toxicity stress							LBI		Grain yield (tons/ha)		Response to Fe-toxicity
		PH	DF	TW	PL	GN	PN	Grain-Fe content					
									toxic Field	Hydroponics	Normal	Fe-toxicity stress	
1	Sankaribako	108	108	25.96	22	78.75	6.7	1.3325	3.5	4	2.55	2.71	MR
2	Kalakrushna	99	99	14.9	24	150.32	6.85	1.2925	4.75	7	4.86	2.15	MS
3	Assamchudi	98	98	24.855	24.35	111.25	6.6	0.9675	5	5	4.85	2.82	MR
4	Nini	96	96	20.155	27	104.05	7.6	1.8175	6.25	8	3.75	1.65	S
5	Champa	105	105	21.965	20	125.57	7.3	0.8525	6.5	8	3.75	2.32	S
6	Mugei	106	106	19.845	22	75.97	6.05	0.7575	5	4	2.96	1.94	MR
7	Latamahu	100	100	18.955	22	92.4	7.3	1.045	3	7	3.92	1.98	MS
8	GeleiA	97	97	15.43	24	122.57	6	2.3925	4.75	6	3.68	1.97	MS
9	Kalamara	95	95	19.06	22.15	75.97	5.65	0.9	3.5	5	2.65	1.88	MR
10	Veleri	113	113	26.815	26.15	81.6	5.55	1.8325	4.75	4	3.62	3.1	MR
11	Gurumukhi	104	104	26.565	20.5	84.25	6.7	6.7775	5	3	3.25	2.85	R
12	Jubaraj	106	106	15.865	26.35	86.07	8.15	0.57	5.75	3	3.45	2.96	R
13	Dhabalabhuta	106	106	25.435	21.65	88.62	7.25	0.8375	3.5	4	3.65	2.98	MR
14	Bangali	99	99	21.69	20.5	98.07	6.75	8.2025	5.75	8	3.95	2.16	S
15	Dhinkisiali	106	106	20.835	19	88.75	8.3	1.185	3.5	6	3.45	1.75	MS
16	Sagiri	101	101	27.795	23	123.75	7.65	4.255	4.75	5	2.89	1.52	MR
17	Bayabhanda	108	108	19.83	21.5	80.67	9.35	1.2475	4	3	3.41	2.52	R
18	Banda	107	107	23.435	28	107.25	6.35	2.7125	5	3	3.78	2.45	R
19	Hatipanjara	106	106	23.13	23	94.3	9.75	2.1725	4.5	6	3.75	2.23	MS
20	Chudi	107	107	22.195	28.5	128.65	6.55	0.8	6.5	7	3.24	2.45	MS
21	Jalpaya	103	103	16.83	23	104.47	6.55	4.9575	3.5	3	3.62	3.12	R
22	Kakiri	103	103	24.435	23.5	103	5.75	1.285	5.25	5	2.89	2.18	MR
23	Ratanmali	106	106	18.79	26	125.67	8.7	0.605	2.75	4	3.12	2.63	MR
24	Dhusura	100	100	23.775	27	82.15	6.45	1.0455	2.25	2	4.34	3.73	R
25	Umarcudi	99	99	20.79	26	125.07	7.25	1.6675	4.5	3	3.24	2.67	R
26	Nilarpati	109	109	28.385	24.85	102.45	6.6	1.7825	4.25	5	3.12	2.97	MR
27	Anu	98	98	12.2	21	147.95	6.85	0.69	5.75	5	3.26	2.51	MR
28	Madia	99	99	23.84	26	106.82	7.1	1.5025	7.25	7	3.56	2.13	MS
29	Ramakrushanabilash	100	100	14.835	24.5	136.8	7.7	0.7025	4.5	5	3.98	3.23	MR
30	GeleiB	105	105	16.825	19.5	133	7.8	1.265	3	3	3.86	2.94	R
31	Sunapani	113	113	22.36	26	119.35	6.7	0.83	3.25	4	5.21	4.73	MR
32	Jabaphula	106	106	24.15	24.5	110.85	5.5	4.9575	4.5	3	2.82	2.42	R
33	Juiphula	103	103	14.21	23.5	147.25	7.25	4.0325	4.25	3	2.93	2.45	R
34	Karpurakranti	104	104	13.54	23.15	108.85	7	1.5675	6.5	6	3.25	1.52	MS
35	Ranisaheba	104	104	18.91	21	129.32	8.05	3.3925	3.75	5	3.65	2.83	MR
36	Mahipal	98	98	19.16	24.5	190.57	6.55	0.532	6.75	5	4.85	4.23	MR
37	Pipalbasa	102	102	24.8	22.15	69	5.2	2.1125	6.75	5	2.35	1.46	MR
38	Jaiphula	98	98	12.095	27.5	94.5	4.95	0.7525	3.25	4	2.54	1.32	MR
39	Mayurkantha	103	103	23.82	24	103.675	7.1	0.865	5.5	7	3.65	2.86	MS
40	Champeisiali	107	107	27.525	23.5	96.375	6.1	0.61	4.75	3	3.75	3.35	R
41	Nalijagannath	107	107	21.695	19.5	123.47	6.3	4.225	6.25	4	3.87	2.33	MS
42	Khandasagar	100	100	12.44	25	77.95	5.8	6.345	5.5	4	2.98	1.77	MS
43	Punjabniswarna	106	106	28.39	29.5	93.475	5.8	2.625	4.5	5	2.45	1.85	MR
44	Kusuma	98	98	19.835	21.5	118.65	5.35	0.665	3	4	2.84	2.34	MR
45	Kendrajhali	105	105	17.99	23	121.02	5.3	0.67	4.75	3	2.54	2.06	R
46	Biridibankoi	114	114	22.755	21.5	103.82	7.8	5.1	4	5	3.45	3.25	MR
47	Jagabalia	113	113	26.19	20	138.5	7.35	1.3675	4.5	4	4.45	3.18	MR
48	Basapatri	97	97	21.905	22	106.42	7.4	0.755	5.75	2	2.95	2.32	R
49	Kalaheera	108	108	26.765	21.85	116.22	7.9	0.755	3	4	3.74	3.55	MR
50	Budidhan	97	97	12.09	27.85	113.15	6.4	1.793	2.75	8	2.96	1.35	S
51	Karpuragundi	104	104	12.13	24	126.3	6.6	0.915	5	6	2.46	1.95	MS
52	Dhoiamadhoi	104	104	25.25	27.5	103.75	5.75	1.055	5.75	5	2.84	2.34	MR
53	Bagadachinamala	104	104	12.69	26	100.65	5.8	0.57	4.25	6	3.68	2.12	MS
54	Kaniara	104	104	13.245	21	101.25	6.55	0.5775	2.75	5	2.55	1.98	MR
55	Rasapanjari	99	99	29.54	26	128.9	5.15	0.635	4.25	3	3.71	3.36	R
56	Mayurachulia	106	106	14.36	19	165.12	5.85	2.455	4.25	7	3.65	2.52	MS
57	Madhabi	108	108	26.485	22	126.55	5.7	1.145	5.75	6	3.12	2.08	MS
58	Rangasiuli	104	104	22.3	28.5	118.3	5.65	2.035	4.5	4	2.98	2.36	MR
59	Saluagaja	102	102	15.055	25	141.12	6.3	0.6725	6.25	3	2.56	2.28	R
60	Bishnupriya	109	109	24.645	23	127.9	7.4	1.7025	4.75	3	3.45	3.04	R
61	Tikimahsuri	105	105	13.7	28	126.17	7.7	0.42	4.5	3	3.24	2.15	R
62	Jungajhata	103	103	23.59	27	112.57	7.85	0.6075	4	3	2.96	2.54	R
63	Asinasita	103	103	12.96	19	104.75	7.85	2.92	4	3	2.98	2.61	R
64	Sankarachini	100	100	26.855	23.5	100.3	6.35	0.49	3.25	4	2.52	1.89	MR
65	Kalajeera	109	109	14.66	22	155.1	8.35	15.2	3.5	4	3.12	2.65	MR
66	Bsudha	104	104	18.97	24	136	5.3	9.4425	6.5	4	2.74	2.06	MR
67	Basudha	107	107	14.74	21	149.85	5.8	1.7875	3	5	2.96	2.75	MR
68	Kabir	107	107	22.235	21.5	119.9	5.95	6.015	2.75	3	2.65	2.15	R
69	Tulasibasa	102	102	22.32	25.65	116.47	5.85	0.7	3	4	2.95	2.55	MR
70	Nalikalma	105	105	23.74	25	142.1	6.4	0.73	2.75	3	3.05	2.82	R
71	Bhangar	106	106	12.51	22.5	124.72	7.8	8.905	4.25	4	2.68	2.16	MR
72	Malata	108	108	22.03	20	110.75	7.75	4.115	5.25	5	3.65	2.45	MR
73	Gobindabhog	104	104	12.76	21	166.4	6.9	0.9945	4.75	4	3.78	2.76	MR
74	Latachaunri	103	103	24.57	24.5	147.33	6.6	1.615	2.75	2	2.98	2.47	R
75	Agnisar	105	105	21.74	19	109.47	5.45	1.034	5.75	8	3.78	2.17	S
76	Luna	102	102	27.99	23	102.47	6.4	1.7925	4.25	4	3.45	2.76	MR
77	Sebati	100	100	23.6	23.5	111	5.8	0.83	5.75	8	3.65	1.52	S
78	Nadalghanta	109	109	23.71	21.5	119.9	6.15	0.91	4	4	3.45	3.13	MR
79	Bhutmundi	110	110	25.13	20	103.07	5.3	2.0675	3	3	2.68	2.42	R
80	Jata	102	102	23.54	22.5	114.57	6.5	0.6175	5.25	6	3.12	2.24	MS
81	Sarubhajana	104	104	24.265	21	122.4	5.6	0.775	3.75	3	2.65	2.25	R
82	Tulasimali	103	103	26.15	23	106.475	5.7	1.205	4.5	3	2.95	2.65	R
83	Abhiram	97	97	18.74	21.85	111.25	5.15	0.9425	7.5	6	3.65	2.73	MS
84	Pateni	105	105	18.53	24	127.5	5.95	1.9375	3	5	3.65	3.25	MR
85	Ahirman	106	106	27.1	21	86.87	6.2	1.0175	3.75	5	2.56	1.95	MR
86	Malliphulajhuli	99	99	19.65	22.5	105.45	7.1	1.34	4.5	4	2.85	2.41	MR
87	Makarkanda	104	104	26.465	25	102.4	5.9	0.7175	4	3	2.85	2.54	R
88	Bharati	98	98	18.58	19.5	126.3	5.55	1.315	5.25	4	3.12	2.86	MR
89	Khajurikandi	98	98	13.76	23	115	6.15	2.1925	3	5	2.25	1.85	MR
90	Sapri	100	100	24.35	26.5	107.2	6.4	2.2025	3	5	3.42	3.04	MR
91	Dhoiabankoi	107	107	23.42	21.5	98	5.5	2.065	4	3	3.12	2.89	R
92	Nalibaunsagaja	103	103	27	21.5	115.6	8.1	0.8775	4.5	6	3.45	2.55	MS
93	Malabati	109	109	23.32	21	106.2	7.6	1.24	6.25	8	3.84	2.18	S
94	Kalamulia	110	110	25.39	22	97.9	6.15	0.77	5	2	3.45	2.98	R
95	Nikipakhia	97	97	18.45	25	117.1	6.15	1.6125	5.5	7	3.14	1.98	MS
96	Saraswati	111	111	28.48	24	117.2	6.1	0.6275	4.75	5	4.64	3.55	MR
97	Jhilli	104	104	20.34	25	125.0	9.5	23.6925	3.75	5	3.25	2.51	MR
98	Budhamanda	110	110	26.83	24.5	109.2	6.45	0.8975	3	4	2.85	2.64	MR
99	Hunder	105	105	18.6	23	115.5	5.4	2.2125	5	5	2.95	2.47	MR
100	Haribhog	106	106	18.025	23	98.4	9.3	1.14	5	2	3.65	3.27	R
101	Labangalata	111	111	17.91	21	184.2	7.25	1.08	4	5	3.85	3.25	MR
102	Korkaili	62	62	24.31	22.5	129	5.8	2.8925	6.25	8	3.56	1.96	S
103	Matiakhoja	107	107	26.715	25.5	102	7.05	1.565	5.5	5	3.45	2.68	MR
104	Kusumkunda	95	595	26.345	22	102.9	6.35	1.0125	4.5	6	3.85	2.46	MS
105	Padmakesari	109	109	18.55	22.5	124.3	7.3	2.8225	2.5	3	3.85	3.53	R
106	Kanchan	120	120	21.7	20.85	151.7	8.1	0.635	4	4	5.21	4.42	MR
107	Khajara	107	107	28.7	26.5	128.3	7.05	0.6	3.75	3	3.34	2.92	R
108	Rambha	119	119	21.98	24.5	133.5	7.45	0.7725	5.5	3	4.62	3.72	MR
109	Mahalaxmi	113	113	18.96	21.85	189	8.4	6.815	6.25	5	5.36	4.15	MS
110	Harisankar	109	109	17.35	22	123.3	5.45	29.505	5.5	2	3.65	3.38	R
111	Dimapur	108	108	17.925	18	172.8	6.1	1.71	3	4	3.25	2.58	MR
112	Sreebalaram	111	111	18.485	25.5	159.6	6.35	0	3.75	2	4.85	4.59	MR
113	Dhanashree	111	111	18.79	19	122.2	6.55	1.955	4.75	3	3.56	3.28	MR
114	Khndiratnachudi	109	109	26.66	26	107.3	6.45	13.1375	4.5	3	2.95	2.69	MR
115	Ruksal	115	115	24.575	25	153	5.6	1.76	5	3	3.25	2.85	MR
116	Jagannath	113	113	20.5	19.5	161.8	6.55	1.0475	6.25	7	5.45	2.86	MS
117	Manika	110	110	17.6	19	156.6	6.8	0.7925	6	4	5.12	3.13	MR
118	Urbashi	116	116	17.96	26	118.3	6.4	13.4475	5.25	8	5.36	2.75	S
119	Salivahan	120	120	15.55	21.15	135.8	7.6	0.745	4	5	5.16	2.28	MR
LSD_5%_		10.7	9.87	1.95	1.81	13.87	0.712	0.49	-	-	4.31	3.47	
CV%		3.57	4.13	6.24	5.42	12.53	9.78	6.49	-	-	9.86	13.17	

PH: Plant height (cm); DF: Days to 50% flowering; TW: Seed 100-seed weight (g); PL: Panicle length (cm); GN: Number of grains/panicle; PN: Number of Panicles/plant and LBI: Leaf bronzing index

### Phenotyping of germplasm lines under Fe-toxicity field

The seeds were sown in the nursery bed and were transplanted in an iron toxicity hotspot field/ sick plot and a normal plot without Fe toxicity at a spacing of 15 cm apart and 20 cm between rows. The screening experiment for Fe-toxicity tolerance was planted by giving 3 lines/ each germplasm in an augmented block design with two checks and seven blocks during wet season, 2016. The shortlisted panel population was planted in a randomized complete block design (RCBD) with three replications per genotype for phenotyping during wet seasons, 2017 and 2018. A good crop was raised by adopting the recommended fertilizers and other practices. The initial Fe level in the sick plot was estimated from five different sampling locations of the plot that ranged between 225–250 ppm [38]. The field was maintained under saturated anaerobic condition. Phenotyping of germplasm lines was performed for days to 50% flowering, plant height, panicle length, number of grains per panicle, 1000-grain weight, grain yield, leaf bronzing index, numbers of tillers/hill and grain-Fe content. These observations were recorded by following Standard Evaluation System of Rice [39] while Fe content was estimated using the energy dispersive x-ray fluorescence spectrophotometer (ED-XRF) X-supreme 8000 using 5g polished rice samples [6]. LBI of each genotype was recorded from three replications. The genotypes were considered as susceptible at a score of 6 to 9; moderately resistant at 4–5; resistant at 1–3 and 0 as immune to Fe- toxicity tolerance.

### *In vitro* screening of panel population for Fe-toxicity tolerance

*In vitro* screening of the panel population containing 119 genotypes was carried out under RGA- cum-phytotron facility of ICAR-NRRI, Cuttack. Screening experiments were conducted in a hydroponic system. The pre-sterilized seeds with 0.1% HgCl_2_ for 3 min followed by heat treatment at 45° C for 6 hrs were germinated in petriplates. Seven days old seedlings were transferred to Yoshida medium, pH 5.0 in different hydroponics container of size 10.5’’x14.5’’x3.0’’ and 5.0”x6.0”x2.0” [30]. Plants were fixed with sponges on a styrofoam. The experiment was conducted with three independent replications each one having six plants of each genotype under the study. The independent replications were carried out at different time points of same rice season keeping other factors constant. Supplied Yoshida medium was replaced after each three days to maintain pH and nutrient composition of medium. A 10 day Fe pulse stress of 1000 ppm (as FeSO4.7H2O) was applied at 4 weeks after moving the plants to hydroponics culture. Same experimental set up for LBI under control and treatment with Fe was maintained with only difference that the control set up was without any Fe stress, whereas the treatment set up was applied with Fe pulse stress of 1000 ppm. As a measure of Fe stress, LBI score ranging from 1 to 9 was assigned to the three youngest fully expanded leaves of each plant on the tenth day of pulse stress following the procedure described in earlier publication [23].

### DNA isolation and molecular characterization

The genotyping work was carried out in the Molecular Breeding Lab-I, Crop Improvement Division, ICAR-NRRI, Cuttack, Odisha. Total genomic DNA was extracted from five week old plants of the rice germplasm lines and varieties following stepwise CTAB protocol [40]. PCR amplification was performed in a Gradient Thermal Cycler (Veriti, Applied BioSciences). The fifty one markers including 47 SSR and 4 reported gene specific markers covering all 12 chromosomes of rice were used in the present study are presented in the S2 Table. The amplified products were loaded in 3% agarose gel containing 0.8 μg/ml ethidium bromide for electrophoresis in 1X TBE buffer (pH 8.0). DNA ladder (50bp) was used to identify the size of the amplicons. The gel was run at 2.5V/cm for 4 hrs and photographed using a Gel Documentation System (SynGene). The data were scored for each genotype-primer combination based on the size of amplified products for co-dominant and presence or absence of dominant markers. The genetic diversity parameters like number of alleles, allele frequency, gene diversity, heterozygosis, inbreeding coefficient (f) and polymorphic information index (PIC) were estimated using the program PowerMarker Ver3.25 [41]. The marker-trait association analysis was carried out taking both generalized linear model (GLM) and mixed linear model (MLM) by using TASSEL5 software [42]. K has been included as a co-variate in the association study. False discovery rate (FDR) and adjusted *p* values (*q* values) were estimated using the standard procedure followed in earlier publication [44, 45]. The detailed protocols followed for population structure, analysis of molecular variance (AMOVA) and cluster analysis were performed using STRUCTURE 2.3.6, GenAlEx 6.5 and Darwin 5 softwares, respectively described in earlier publications [43–47]. The parameter set used in the structure program as ‘possibility of admixture and allele frequency correlated’ having burn-in period of 2,00,000 and Markov Chain Monte Carlo replications of 2,00,000. K value was taken from 1 to 10 having 10 times repetition each. The most probable number of subpopulations present in the panel population was determined by taking the peak value of delta K. The deviation from Hardy-Weinberg expectation within a population (F_IS_), between sub-populations (F_ST_) and across the whole population (F_IT_) was estimated by analysis of molecular variance. Unweighted neighbor joining method was followed for constructing unrooted tree based on dissimilarity matrix of Nei coefficient. Bootstrap value of 1000 had been taken while constructing the tree.

## Results

### Phenotyping of germplasm lines for Fe-toxicity tolerance

**Fig 1:**
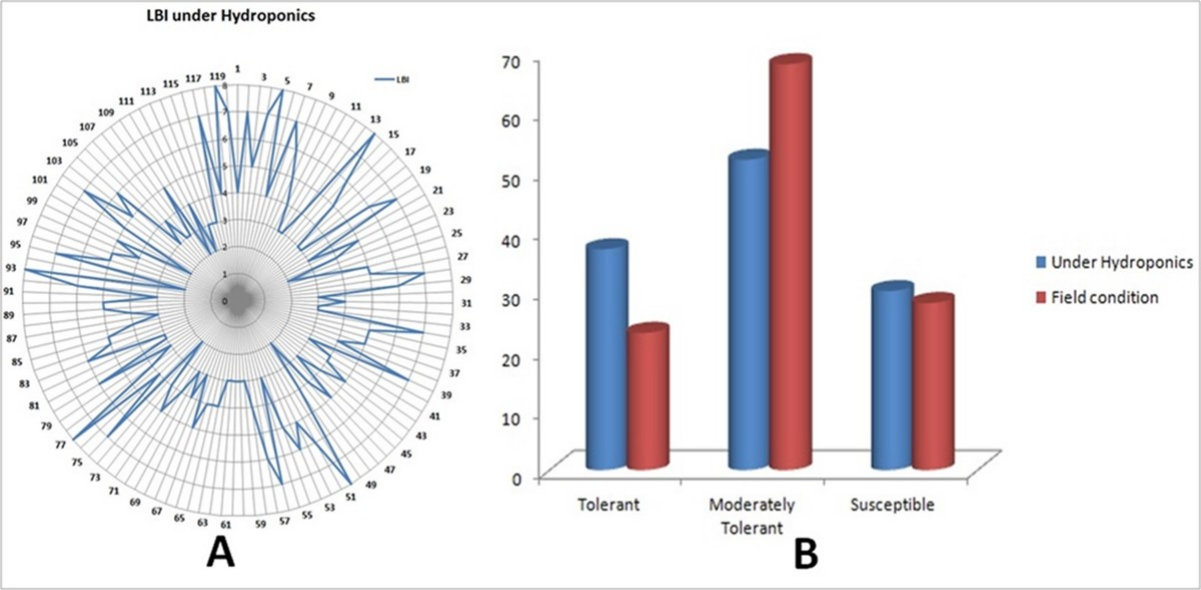
Leaf bronzing index of the 119 germplasm lines. (A) LBI of the 119 germplasm lines under hydroponics condition; (B) Frequency distribution of the LBI in the 119 germplasm lines under field and hydroponics conditions.

Screening of 352 germplasm lines for Fe-toxicity tolerance was performed in the Fe-toxicity sick plot for constitution of a panel population. The susceptible check variety showed high score of 9 for leaf bronzing symptoms under the field evaluation. A total of 153 germplasm lines were tolerant (1–3 Score), 103 moderately tolerant (4–5 score) and 96 were observed to be susceptible to iron toxicity (S1 Table; S1 Fig). A panel population containing 119 genotypes representing all phenotypic classes was constituted for genotyping and phenotyping (Table 1). The shortlisted genotypes were transplanted under both sick plot and hydroponic conditions. The leaf bronzing index scores of the genotypes varied from 1.0 to 9.0 under the Fe-toxicity field screening and controlled condition under hydroponics culture. The number of genotypes showing LBI score of 1 to 3 were 23 under field evaluation while 37 in hydroponics screening (Table 1; Fig 1). The LBI score of 3.0 to 5.0 were observed in 68 and 56 genotypes under field and hydroponics screening, respectively. However, the susceptible germplasm lines showing 6–9 score were 28 under field evaluation and 32 in hydroponics screening. The genotypes producing higher yield under normal condition and at par yield under stress are considered as promising genotypes. Evaluation of 119 genotypes under normal and Fe-stressed condition revealed the presence of promising landraces showing both high yield and tolerance to the stress (Table 1). These desirable genotypes namely Dhusara, Champeisali, Rasapanjari, Padmakeshari, Harisankar, Sreebalaram, Sunapani and Mahipal may be recommended for Fe-toxicity areas or as the donor parent in the breeding programs. The frequency of genotypes showing poor, moderate and high tolerance to the stress is depicted in the bar diagram (Fig 1).

**Fig 2:**
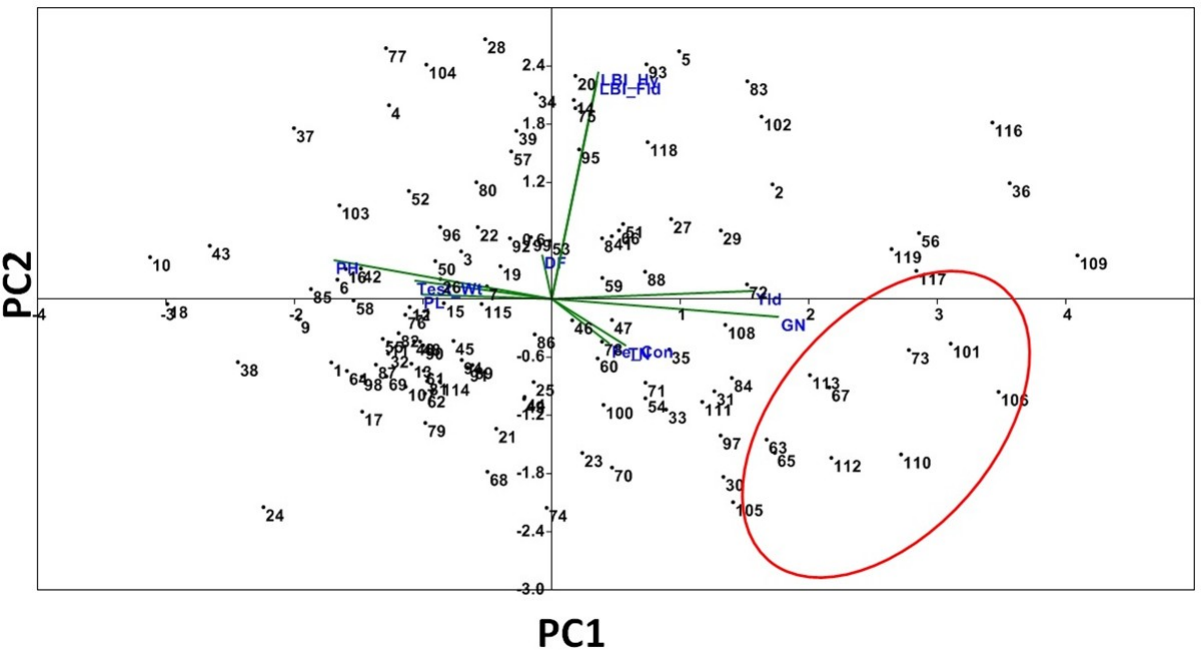
Genotype-by-trait biplot graph depicting genotypes in two main principal components for 9 traits. LBI: Leaf bronzing index; yld: Grain yield (tons/ha); GN: Number of grains/panicle; PH: Plant height (cm); TN: Number of tillers/plant; DFF: Days to 50% flowering; TW: 1000-seed test weight (g); GN: Number of grains/panicle; Fe C: Iron content in grain (ppm). The spot numbers in the graph denotes the genotypes serial number as enlisted in Table 1.

Genotype-by-trait biplot analysis involving 2 principal components was constructed to identify the stable genotypes and to know the extent of variation among the genotypes present in the panel population. The first principal component (PC) accounted for 22% with eigen value of 2.2. The PC2 exhibited 13.5% variance with an eigen effect of 1.35. The genotypes showing high score for leaf bronzing index were in the same quadrant revealing similar phenotypes to the toxicity response. The encircled area in the quadrant accommodates the desirable genotypes with low bronzing scores and better yield (Fig 2). The quadrant 4 accommodates all the susceptible genotypes showing low tolerance to soil Fe-toxicity tolerance (Fig 2).

### Genetic diversity

The molecular diversity available in the panel population for iron toxicity tolerance study containing 119 germplasm lines was estimated by employing 51 molecular markers (S2 and S3 Tables). Wide variation in alleles was observed showing a range of 70bp to 380bp. The variation for major allele frequency was also found to be high ranging from 0.289 (RM206) to 1.000 (RM556, Loc_Os01g49710, Loc_Os01g49720) with a mean value of 0.661. A moderate molecular diversity was observed in the panel population as the mean polymorphic information content (PIC) of primers was 0.372. A maximum PIC value of 0.697 was estimated by the marker RM206. The average gene diversity in the panel population was 0.4564 based on the diversity estimated by using 51 markers. The maximum gene diversity (0.745) was detected by RM206 while the minimum gene diversity (0.00) was obtained by using RM556, Loc_Os01g49710 and Loc_Os01g49720 in the 119 rice genotypes present in the panel (Table 2).

**Table 2:** Analysis of molecular variance (AMOVA) of the sub-populations of panel population at K = 3 for Fe-toxicity tolerance in rice of 119 genotypes.

Source of variation	AMOVA for the three sub-populations at K = 3			
	df.	Mean sum of squares	Variance components	Percentage variation
Among populations	2	145.1	1.704	11
Among individuals (accessions) within population	116	23.2	9.981	67
Within individuals (accessions)	119	3.3	3.277	22
Total	237	3376	14.962	100
**F-Statistics**	**Value**	**P-value**		
F_ST_	0.114	0.001		
F_IS_	0.753	0.001		
F_IT_	0.781	0.001		
F_ST_ max.	0.482			
F’_ST_	0.237			

### Genetic structure analysis

**Fig 3:**
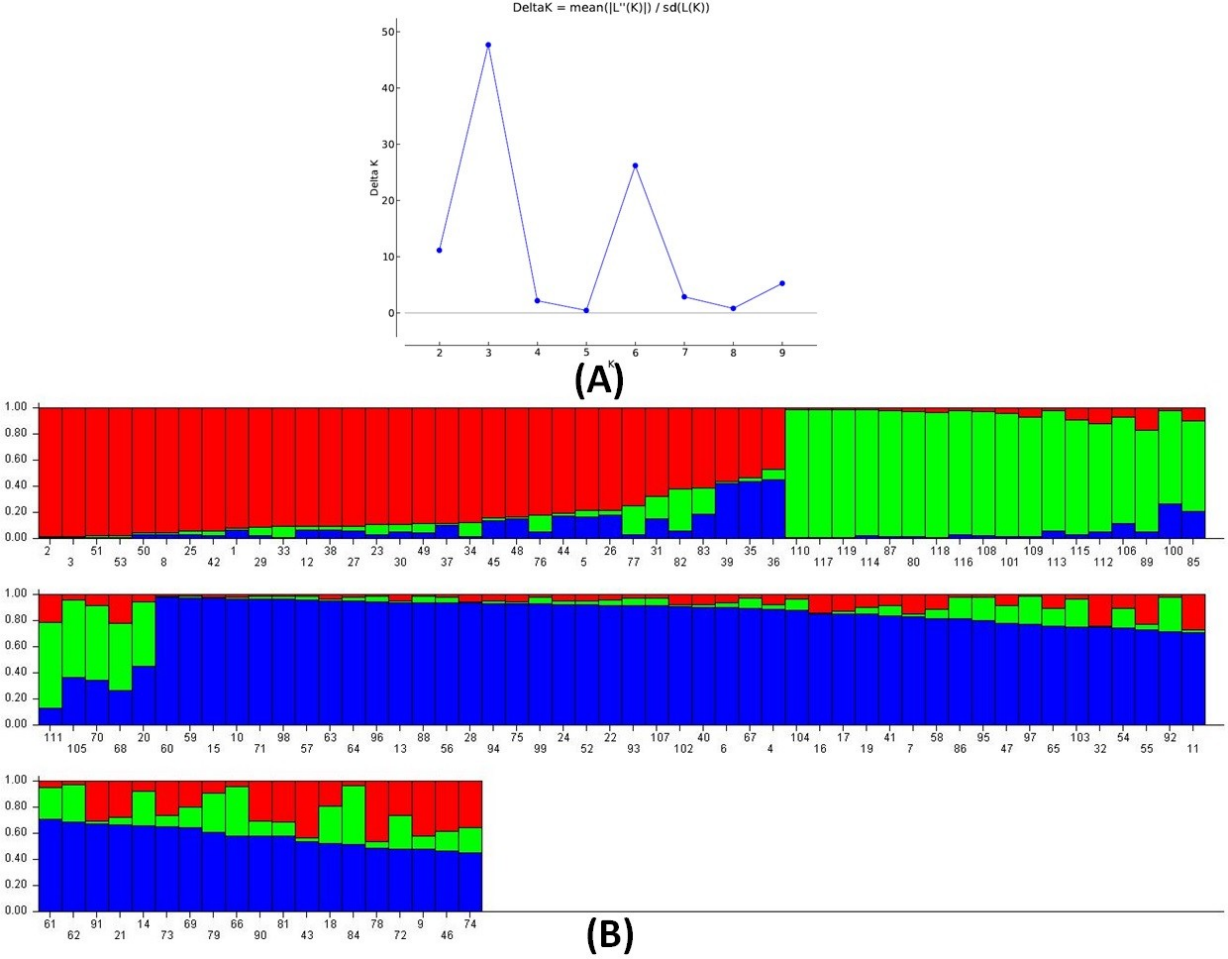
(A) Graph generated by ploting delta K vs. K for determination of peak value and (B) the genetic structure groups obtained for the studied panel population and sorted as per the group. The numbers in the figure denotes the genotypes serial number as enlisted in Table 1.

**Fig 4:**
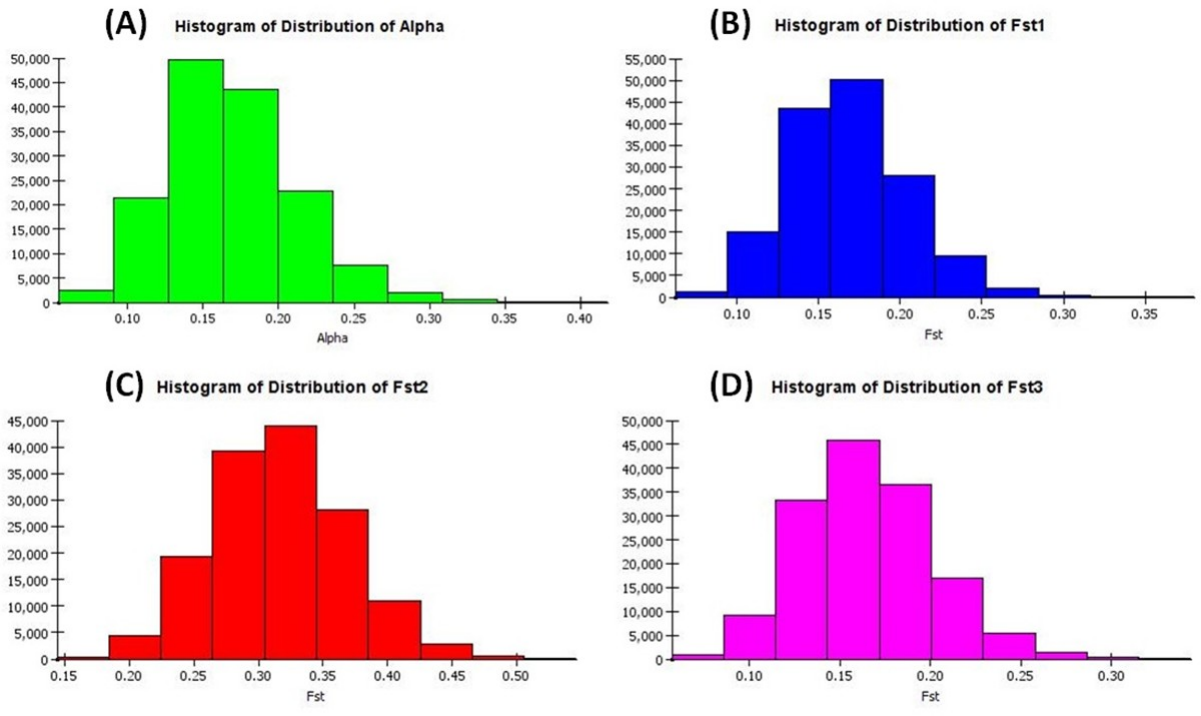
Distribution of (A) Alpha value in panel (B) F_st_ values obtained for subpopulation 1; (C) Fst values obtained for subpopulation 2 and (D) F_st_ values obtained for subpopulation 3.

The presence of genetic variation for the desired traits in the population is essential for improvement of the trait through breeding. The information generated from structure analysis is much useful to the plant breeders for enhancement of various traits including Fe-toxicity tolerance in breeding program. The STRUCTURE software classified the panel population into 3 genetic groups. These classes are obtained based on the peak value of the K and ΔK plot which showed the value at K = 3 (Fig 3; S4 Table). The proportion of membership (overall) in each cluster was 0.296, 0.229 and 0.475 in subpopulation 1, subpopulation 2 and subpopulation 3, respectively. The fixation index (F_st_) values were 0.1687, 0.3162 and 0.1651 for subpopulation 1, 2 and 3, respectively. The allele-frequency divergence based on net nucleotide distance showed a value of 0.0979 in subpopulation 1 and 2; 0.0715 for subpopulation 1 and 3 while 0.0857 for subpopulation 2 and 3. The expected heterozygosity (average distances) between individuals in the subpopulation 1, subpopulation 2 and subpopulation 3 showed 0.4155, 0.3402 and 0.3848 distance, respectively. A fair degree of correspondence was observed for Fe-toxicity tolerance in the genotypes and subpopulation groups in the studied panel (Fig 3; S4 Table). Thus, the peak of ΔK at K = 3 was utilized for the analysis. Most of the Fe-toxicity tolerant genotypes were in the subpopulation 1 while moderately tolerant types were observed under subpopulation 3. The alpha value detected by structure analysis from the panel population was 0.1686 indicating a lower value of alpha at K = 3. A leptokurtic distribution curve was observed for alpha-value and for 3 subpopulations at K = 3 (Fig 4).

### Clustering of germplasm lines based on leaf bronzing index and agro-morphologic traits

**Fig 5:**
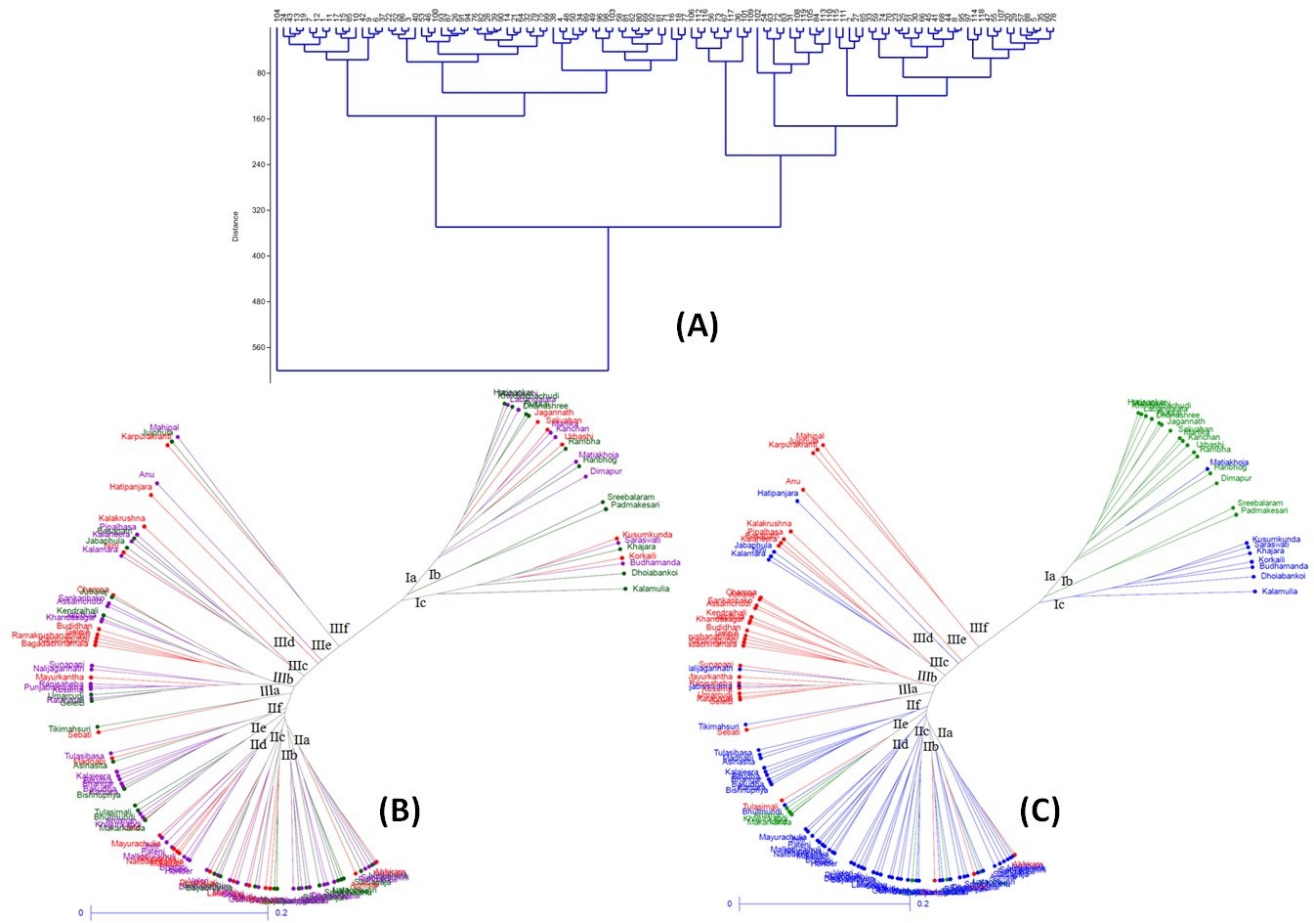
Clustering of 119 genotypes. (A) Ward’s Cluster diagram based on the nine morphological descriptors; (B) Dendrogram based on the genotypic data: green, violet, and red colors indicate tolerant, moderately tolerant, and susceptible genotypes respectively based on LBI in hydroponics condition; (C) Dendrogram based on the genotypic data: genotypes in red, green, and blue colors indicate membership in SP1, SP2, and SP3 based on population structure analysis.

The genetic relatedness among 119 germplasm lines was investigated by generating a dendrogram using Ward’s clustering approach (Fig 5). The genotypes were clearly differentiated into various groups and subgroups based on the 9 descriptors (Fig 5A). The cluster analysis using Ward’s method showed two major clusters of which one consisted sixty three genotypes and second one included rest 56 genotypes. The first major cluster was again sub divided into two sub-clusters. The tolerant and moderately tolerant genotypes Dhusura, Punjabni swarna, Dhabalabhuta, Sankaribako, Gurumukhi, Bayabhanda, Ahirman, Veleri, Khandasagar, Kalamara, and Pipalbasa were grouped together in one sub-cluster.

The cluster analysis based on genotyping with 51 molecular markers showed three major clusters consisting of 24, 60 and 35 genotypes (Fig 5B). Cluster I, II and III are again sub divided into 3, 5 and 6 sub-clusters, respectively. The tree constructed based on genotyping data was colored according to the phenotypic classification of the panel population with respect to their response to Fe toxicity tolerance under controlled condition (Fig 5B) and the sub-population groups according to structure analysis (Fig 5C). The major cluster I and II consisted of SP2 and SP3 category genotypes. The cluster I included majority of the SP2 type genotypes (sub-cluster Ia and b) and few members of SP3 group, whereas, the cluster II included majority of SP3 type genotypes. SP1 category genotypes were accommodated in the cluster III. When response of the genotypes for Fe toxicity tolerance in terms of LBI was compared with the grouping pattern based on 51 marker data, the tolerant and moderately tolerant lines were grouped together forming distinct sub-clusters. The major cluster I included 11 tolerant, 8 moderately tolerant genotypes along with only five susceptible ones, namely, Jagannath, Salivahan, Urbashi, Korkaili and Kusumkunda. Similarly, cluster II also included majority of the tolerant (18) and moderately tolerant (29) ones with 13 susceptible genotypes having LBI score 6 to 9. The sub-cluster IIb comprised only tolerant and moderately tolerant lines namely, Champeisiali, Kabir, Rasapanjari, Sarubhajana, Nalikalma, Latachaunri, Nilarpati, Nadalghanta, Malata, Luna, Dhoiamadhoi and Biridibankoi, whereas, sub-cluster IIa, IIe and IIf included only tolerant and moderately tolerant genotypes along with either one or two susceptible genotypes.

### Analysis of molecular variance (AMOVA) and LD decay plot

The analysis of molecular variance (AMOVA) showed genetic variations between and within the sub-populations at K = 3 (Table 2). The genetic variations between and within the three sub-populations (K = 3) was 11% among the populations, 67% among individuals and 22% variation within individuals in the panel population. Wright’s F statistic was used to calculate the deviation from Hardy-Weinberg’s prediction. The F_IS_ and F_IT_ values for all the 51 loci were 0.753 and 0.781, whereas F_st_ was 0.114 among populations at three sub-populations analysis (Table 2). The F_st_ values at K = 3 could discriminate the sub-populations from each other revealing differences among themselves. A moderate linearized F_st_ value was estimated for the sub-populations. The F_st_ values of each sub-population and their distribution pattern showed a clear differentiation among the sub-populations from each other (Fig 4).

**Fig 6:**
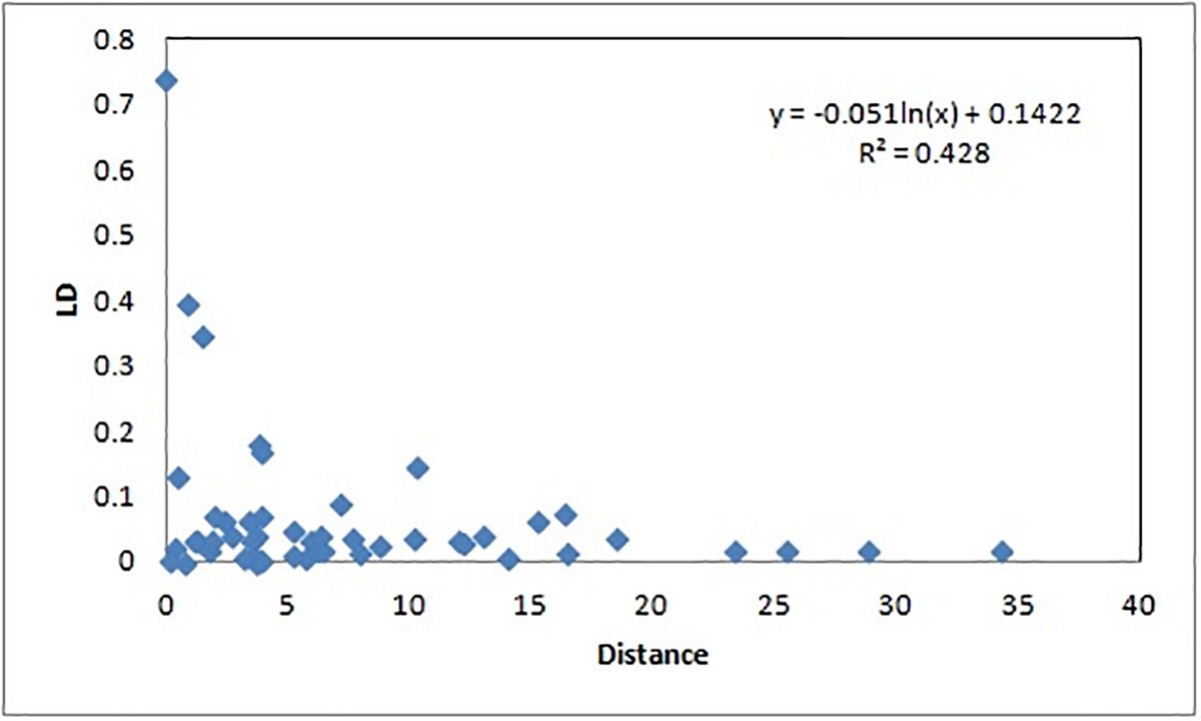
Linkage disequilibrium (LD) decay (*r*^2^) curve plotted against the physical distance (base pairs) between pairs of loci on chromosomes in rice. The decay started in million bp estimated by taking 95^th^ percentile of the distribution of *r*^2^ for all unlinked loci.

The marker–trait associations were detected based on the presence of LD in the population. Therefore, the LD decay rate is important factor to determine the durability and improvement of an associated trait in the population. The Syntenic *r*2 was used to plot the LD decay of the population against the physical distance in million base pair. The plot showed a sharp decline in the decay of disequilibrium for the linked markers at 1–2 mega base pair and thereafter observed a very slow and gradual decay for attaining equilibrium (Fig 6).

### Fe-toxicity tolerance and other traits association with the molecular markers

**Fig 7:**
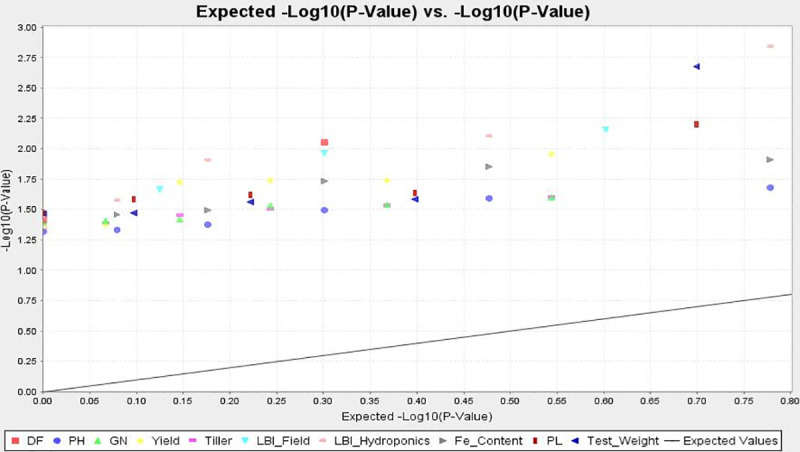
Quantile-quantile plot showing the significantly associated molecular markers with different traits by adopting MLM approach.

A significant association of marker-trait was noticed for few molecular markers with Fe-toxicity tolerance and other related traits in rice using TASSEL5.0. The significant associations were estimated and compared in both GLM and MLM approaches at p<0.05 (Table 3). The r^2^ values varied from 0.04827 to 0.09845 in GLM and 0.04235 to 0.08958 in MLM analysis for LBI at p<0.05. Considering both models at p<0.05, 3, 5, 3, 4, 5 and 4 markers were associated with grain number, yield, tiller number, LBI, grain-Fe content and seed test weight, respectively under Fe-toxicity stress. LBI, an important parameter for Fe-toxicity tolerance was detected to be associated with markers RM471, RM3, RM590 and RM243 by using both GLM and MLM models at p<0.05. RM590, RM574 and RM3412 showed association with grain number by using both the models. *Per se* yield and grain Fe-content showed association with five markers by the models (Table 3). Markers RM31, RM202, OsIRT1 and RM245 were detected to be associated with seed test weight. The QQ plot confirmed the association of markers with all the traits under study (Fig 7).

**Table 3:** Marker-trait association of for the traits leaf bronzing index, grain Fe-content, grain yield and related traits through GLM and MLM approach in rice.

Sl.No.	Trait	Marker	*F Value*	*p Value*	*R*^*2*^	*F Value*	*p Value*	*R*^*2*^
			GLM			MLM		
1	GN	RM590	6.06979	0.01527	0.03927	4.36054	0.03905	0.03765
2	GN	RM574	6.64923	0.01122	0.0428	4.86943	0.02938	0.04205
3	GN	RM3412	10.92977	0.00127	0.06791	8.72845	0.00382	0.07537
4	Yield	RM168	8.87483	0.00355	0.05163	9.72668	0.00231	0.08414
5	Yield	RM5638	6.16787	0.01449	0.03671	4.09431	0.04541	0.03542
6	Yield	RM7	7.22739	0.00828	0.04263	5.73975	0.01824	0.04965
7	Yield	Loc_Os01g49710	4.19827	0.0428	0.02541	4.24027	0.04179	0.03668
8	Yield	RM7003	5.73302	0.01831	0.03425	6.66442	0.01113	0.05765
9	PN	RM202	7.74569	0.00632	0.06401	4.88842	0.02907	0.04029
10	PN	RM269	8.21342	0.00497	0.06761	7.11616	0.00877	0.05865
11	PN	RM3412	7.4365	0.00742	0.06162	5.16683	0.02493	0.04258
12	LBI_Hydroponics	RM471	13.36185	3.93E-04	0.09845	10.69169	0.00143	0.08958
13	LBI_Hydroponics	RM3	6.17512	0.01443	0.04827	5.05458	0.02652	0.04235
14	LBI_Hydroponics	RM590	6.74653	0.01065	0.05248	6.46481	0.01237	0.05416
15	LBI_Hydroponics	RM243	6.91119	0.00977	0.05369	7.06995	0.00899	0.05923
16	Fe_Content	RM590	4.75236	0.03135	0.0382	4.29142	0.0406	0.0382
17	Fe_Content	RM1278	5.238	0.02397	0.04193	4.71033	0.03209	0.04193
18	Fe_Content	RM488	7.04156	0.00912	0.05552	6.23629	0.01397	0.05552
19	Fe_Content	RM17	6.43059	0.0126	0.05096	5.72457	0.01839	0.05096
20	Fe_Content	RM517	7.31724	0.0079	0.05756	6.46553	0.01236	0.05756
21	TW	RM31	5.51493	0.02061	0.03245	4.59509	0.03423	0.03142
22	TW	RM202	4.06783	0.0461	0.02423	5.08734	0.02604	0.03479
23	TW	OsIRT1	10.71141	0.00142	0.06035	9.90152	0.00212	0.06771
24	TW	RM245	7.98123	0.0056	0.04599	4.99098	0.02747	0.03413

GN: Number of grains/panicle; PN: Number of Panicles/plant; TW: Seed 100-seed weight (g) and LBI: Leaf bronzing index

All the markers associated with grain number, test seed weight, grain yield, tiller number, grain-Fe content and LBI under Fe toxicity stress were distributed in all 11 chromosomes except chromosome 9. The four markers RM471, RM3, RM590 and RM243 associated with LBI were located on chromosome 4, 6, 10 and 1 at 18.82, 19.49, 23.04 and 7.97Mb positions, respectively.

## Discussion

Majority of the popular rice varieties are susceptible to Fe-toxicity stress. There is a need to identify potential donors and robust markers for incorporation of tolerance trait into the popular varieties through MAS breeding. In the screening results using 352 genotypes, a wide genetic variation was noticed for leaf bronzing scores staring from 1 to 9 (S1 Table). Three phenotypic groups were obtained for the tolerance to the stress in the studied population. The structure analysis also categorized the population into three subpopulations. The principal component analysis distributed the population of the panel as per their LBI and other traits on different spots in the four quadrants (Fig 2). In addition, the Wards and other clustering approaches also differentiated the population into many sub-clusters (Fig 5). Therefore, it is concluded that the panel population used for the study possesses considerable genetic variation for iron toxicity tolerance. Earlier researchers had also confirmed about the existence of genetic variation for Fe-toxicity tolerance in rice [23, 25, 28–31].

Evaluation of 119 genotypes under normal and Fe-stressed condition revealed the presence of genotypes showing high yield and tolerance to the stress. The placements of promising landraces closer to each other in specified areas are also observed in the constructed biplot (Fig 2). These inferences provide clues about the possibility of improving both Fe-toxicity tolerance and grain yield in rice. The improvement of high grain yield with protein content, Fe-content and Zn content in rice was also reported earlier [6, 45]. The presence of various groups and sub-groups in the phenotype-based clustering and allocation of genotypes to different spots on the PCA quadrant revealed the possibility of involvement of multiple genes/QTL responsible for different groups and sub-groups (Figs 2 and 5). The presence of these sub-groups and groups in the population indicates the existence of linkage disequilibrium in the panel population and helped in marker-trait association for Fe-toxicity tolerance detection. Earlier researchers had also reported detection of marker-phenotype association for different complex traits in rice [24, 25, 43–45, 48–54].

A moderate genetic diversity estimate was observed in the panel population. The present investigation on trait based genetic diversity is almost similar to the earlier findings of moderate genetic diversity parameters reported for single trait [44, 45, 55–58]. However, many earlier reports also detected high diversity parameters for agro-morphological traits in various rice populations [59–62]. The structure analysis categorized the panel’s genotypes into 3 structure groups. The population was divided into subpopulations based on Fe-toxicity tolerance (Fig 3; S4 Table). The green bar inferred ancestry genotypes were mainly associated with moderate to high tolerance to the stress. Majority of the red bar groups (1^st^ subpopulation) showed moderate tolerance to the stress. The blue bar group (subpopulation 3) were mostly low tolerance to Fe-toxicity stress. Thus, structure analysis at the 1^st^ peak at K = 3 categorized the population into 3 subgroups and majority members from each group showed correspondence with tolerance level to the stress. A low alpha value (α = 0.1955) was observed from the structure analysis revealed a common primary ancestor for F-toxicity tolerance of the detected genes. During the course of evolution, the subpopulations with admix genotype might have occurred through natural introgression and further development of many admix type in the population. The inferred ancestry obtained from structure analysis revealed different admix types and provided clues for presence of QTL showing small effects in the individual subpopulations. These small effect QTL need to be pooled together in a single background for imparting greater level of tolerance to the stress which is possible through molecular breeding. Previous research reports on marker-trait studies had also suggested stacking of QTL/gene(s) for enhancement of target traits [43–45, 61].

The F_st_ values and their distribution patterns of the subpopulations at K = 3 were different which provided clue for the subpopulations were different from each other. The shortlisted lines showed clear genetic difference according to within and between subpopulations F_st_ values. Selection of parental line from a population possessing higher F_st_ value is expected to show better chance of obtaining progenies with Fe-toxicity tolerance in recombination breeding. Therefore, efforts need to be given to pyramid the QTL controlling iron toxicity tolerance from different populations resulting in higher tolerance in the progenies. Similar type of opinion were also reported by earlier workers for increasing grain protein content, high and low temperature tress tolerance and grain yield in rice [43–45, 63].

Association of 51 markers with Fe toxicity tolerance for leaf bronzing index under controlled condition in hydroponics culture showed four markers *viz*., RM243, RM590, RM3 and RM471 to be significantly associated by analyzing with both GLM and MLM models. RM243 is located on chromosome 1 at 7.97 Mb position. This result corroborates with the results of [27], where they reported a QTL flanked by markers C955-C885. Hence, this reported QTL is validated in the present study. There is only one marker on chromosome 10 named RM271 reported for LBI by [64]. We detected RM590 to be significantly associated with LBI explaining >5% PV. But this marker is located at 23.04 Mb position which is away from 16.6 Mb position of RM271. Hence, this QTL is considered to be novel and named as *qFeTox10*.*1*. Similarly, two QTL *qFeTox 4*.*1* and *qFeTox 4*.*2* were reported by [28] and one QTL region flanked by markers RM252 and RM451 in the region reported by [64]. These QTL are located around 2–3 Mb, 5–6 Mb and 25–28 Mb, whereas in our study the marker RM471 explaining around 9% PV is located at 18.8 Mb position. So, this is considered novel for Fe toxicity tolerance and termed as *qFeTox 4*.*3*. The literature survey for QTL on chromosome 6 governing Fe toxicity tolerance in terms of LBI and related indirect traits, showed only one region at 10–10.8Mb position controlling traits like shoot fresh weight, shoot dry weight, root dry weight and Fe content under Fe toxicity stress by [65]. In the current study, we detected RM3 positioned at 19.49Mb on chromosome 6 having >4% of PV, which is different from the earlier reports. This novel QTL is designated as *qFeTox 6*.*1*.

One significant correlation is observed in the present study that many QTL for Fe toxicity tolerance and Fe content in rice grain are co-localized. Two novel QTL detected in the present study, *qFeTox6*.*1* is co-localized with the QTL *qFe6*.*1* and *qFe6*.*2*, while *qFeTox10*.*1* with QTL, *qFe10*.*1* [66, 67]. This finding suggests that the traits namely grain-Fe content used for Fe biofortification in rice grain and Fe toxicity tolerance may share some common pathway for channelization of Fe. This needs more detailed study for confirmation. Marker RM590 was associated with Fe-toxicity tolerance and grain number detected by both GLM and MLM approaches. Thus, it may be inferred that grain number and Fe-toxicity tolerance may co-inherit and hence grain yield and Fe-toxicity tolerance may be improved simultaneously.

## Conclusions

A wide genetic variation for Fe-toxicity tolerance was observed in the studied panel population. Principal component and clustering analyses distributed the genotypes into various spots and clusters based on LBI and other traits. A moderate level of genetic diversity was estimated from the population by using 51 molecular markers. Linkage disequilibrium was detected for Fe toxicity tolerance in the panel population. The population was classified into three genetic structure groups. These structure groups corresponded fairly with the Fe-toxicity tolerance in the panel population. The marker-trait association analysis showed association of Fe toxicity tolerance, grain-Fe content and yield component traits using both GLM and MLM models analyzed by TASSEL 5 software. LBI showed significant associations with RM471, RM3, RM590 and RM243 by using both GLM and MLM models. Three novel QTL controlling Fe-toxicity tolerance were detected and designated as *qFeTox4*.*3*, *qFeTox6*.*1* and *qFeTox10*.*1* on chromosome 4, 6 and 10, respectively. A QTL controlling the trait in markers interval of C955-C885 on chromosome 1 reported earlier is validated in the present study. The analysis also showed the co-localization of QTL *qFeTox 6*.*1* with the grain-Fe controlling QTL *qFe6*.*1* and *qFe6*.*2* on chromosome 6 while, *qFeTox10*.*1* was co-localized with *qFe10*.*1* on chromosome 10. The co-localization of the QTL controlling Fe toxicity tolerance with grain Fe content QTLs used for Fe biofortification of rice grain indicates Fe toxicity tolerance and channelization of Fe into rice grain may share some common pathways. It may also be inferred from this study that grain number, Fe-toxicity tolerance and grain Fe content may get co-inherited, hence, these three traits may be improved simultaneously. The Fe-toxicity tolerance QTL detected in this mapping study will be useful in marker-assisted breeding programs.

## Supporting information

S1 Fig Phenotyping for Fe toxicity. (A) Symptoms of Fe toxicity in sick plot taken for the study; (B) Representative picture of plants grown in hydroponic culture; (C) Rice seedlings showing reduction in secondary roots and increased root length with increase in Fe concentration in hydroponic culture; (D) Leaf bronzing symptom in different genotypes under field condition.
(TIF)

S1 Table Screening of 352 rice germplasm lines for Fe-toxicity response evaluated under sick plot during wet season, 2016. (DOCX)

S2 Table List of molecular markers used for association analysis of Fe-toxicity tolerance in rice. (DOCX)

S3 Table Molecular diversity parameter estimates from 119 genotypes using 51 molecular markers. (DOCX)

S4 Table The inferred ancestry value and population structure of the members in a panel containing119 genotypes with their response to Fe-toxicity tolerance. (DOCX)
