# Supplementary figures and images for: Population genetic structure and association mapping for iron toxicity tolerance in rice
Source: PLoS One. 2021 Mar 1;16(3):e0246232. doi: 10.1371/journal.pone.0246232 (PMC7920388; doi:10.1371/journal.pone.0246232)

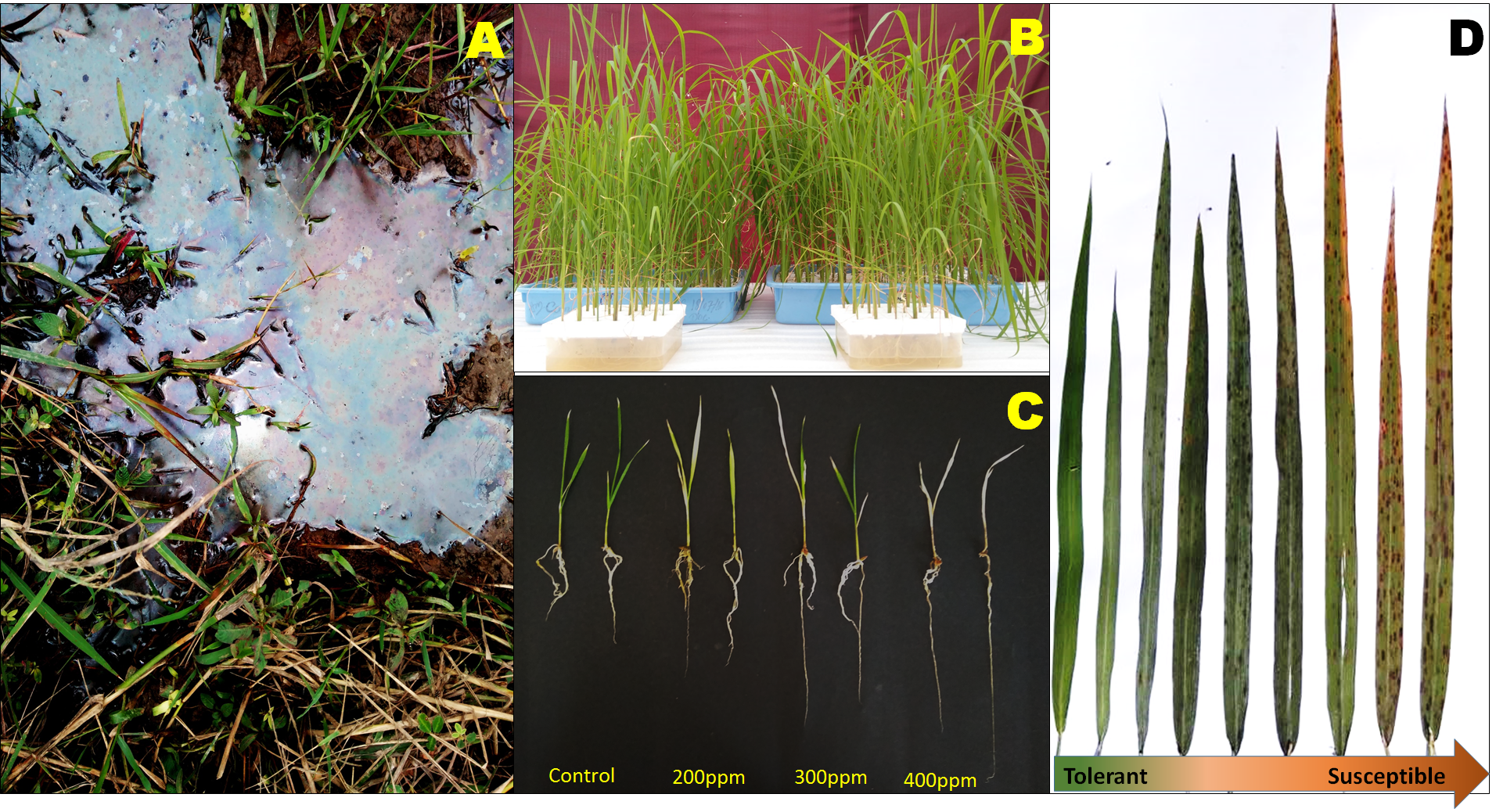

Supplement: S1 Fig — (A) Symptoms of Fe toxicity in sick plot taken for the study; (B) Representative picture of plants grown in hydroponic culture; (C) Rice seedlings showing reduction in secondary roots and increased root length with increase in Fe concentration in hydroponic culture; (D) Leaf bronzing symptom in different genotypes under field condition. (TIF) [file pone.0246232.s001.tif]
