# Supplementary material for: Population genetic structure and association mapping for iron toxicity tolerance in rice
Source: PLoS One. 2021 Mar 1;16(3):e0246232. doi: 10.1371/journal.pone.0246232 (PMC7920388; doi:10.1371/journal.pone.0246232)
Supplement: S1 Table — (DOCX) [file pone.0246232.s002.docx]

**S1 Table**. Screening of 352 rice germplasm lines for Fe-toxicity response evaluated under sick plot during wet season, 2016

| Sl.No | Name of the genotype | LBI score | Sl. No. | Name of the genotype | LBI score |
| --- | --- | --- | --- | --- | --- |
| 1 | Pooja | 7 | 177 | Latachaunri | 1 |
| 2 | Kalakrushna | 7 | 178 | Karangi | 5 |
| 3 | Samba Mahsuri | 7 | 179 | ASD-18 | 9 |
| 4 | Ranjit | 7 | 180 | ASD-16 | 9 |
| 5 | Savitri | 7 | 181 | PM-706 | 9 |
| 6 | Sankaribako | 3 | 182 | AM-10-7 | 9 |
| 7 | Pradhan Dhan | 7 | 183 | Latamahu | 7 |
| 8 | IR44 | 5 | 184 | PM-717 | 9 |
| 9 | Supriya | 7 | 185 | TN1 | 7 |
| 10 | Pathara | 9 | 186 | Nini | 7 |
| 11 | Mugei | 3 | 187 | PTB-10 | 7 |
| 12 | IR36 | 7 | 188 | Mandakini | 7 |
| 13 | Meher | 7 | 189 | Champa | 9 |
| 14 | Kalamara | 5 | 190 | Bangali | 9 |
| 15 | Veleri | 5 | 191 | Lalitgiri | 7 |
| 16 | Gurumukhi | 3 | 192 | GeleiA | 7 |
| 17 | Jubaraj | 3 | 193 | Hatipanjara | 7 |
| 18 | Dhabalabhuta | 5 | 194 | Urbasi | 7 |
| 19 | Mrunalini | 9 | 195 | Chudi | 7 |
| 20 | IR46 | 5 | 196 | Indravati | 7 |
| 21 | Sagiri | 5 | 197 | Manaswini | 7 |
| 22 | Bayabhanda | 3 | 198 | Niligiri | 7 |
| 23 | Banda | 3 | 199 | Birupa | 7 |
| 24 | Jagabandhu | 7 | 200 | Ramachandi | 7 |
| 25 | Bhoi | 7 | 201 | Gouri | 7 |
| 26 | Jalpaya | 3 | 202 | Assamchudi | 5 |
| 27 | Annapurna | 5 | 203 | IR45 | 5 |
| 28 | Ratanmali | 5 | 204 | Dhinkisiali | 5 |
| 29 | Dhusura | 3 | 205 | CR691-47 | 5 |
| 30 | Umarcudi | 3 | 206 | Ramakrushanabilash | 5 |
| 31 | Radhi | 5 | 207 | CR800-1 | 5 |
| 32 | Anu | 5 | 208 | Kakiri | 5 |
| 33 | Madia | 7 | 209 | Sita | 5 |
| 34 | KalingaIII | 5 | 210 | Mahipal | 5 |
| 35 | GeleiB | 3 | 211 | Satabdi | 5 |
| 36 | Karangi | 5 | 212 | Nilarpati | 5 |
| 37 | Jabaphula | 3 | 213 | IR36 | 5 |
| 38 | Juiphula | 3 | 214 | Annada | 5 |
| 39 | Lunisree | 5 | 215 | Karpurakranti | 5 |
| 40 | Ranisaheba | 5 | 216 | Bhuban | 5 |
| 41 | CR816-1 | 5 | 217 | Pipalbasa | 5 |
| 42 | Matangini | 5 | 218 | Sunapani | 5 |
| 43 | Jaiphula | 3 | 219 | IR72 | 5 |
| 44 | Mayurkantha | 7 | 220 | KJT4 | 5 |
| 45 | Ussar | 5 | 221 | Sarala | 5 |
| 46 | Kasturi | 5 | 222 | CR Dhan407 | 3 |
| 47 | Mangala | 5 | 223 | Varshadhan | 3 |
| 48 | Krishna Hamsa | 5 | 224 | Durga | 5 |
| 49 | Kusuma | 3 | 225 | Pant Dhan4 | 5 |
| 50 | Kendrajhali | 3 | 226 | Pant Dhan10 | 5 |
| 51 | Pant dhan 12 | 5 | 227 | Biridibankoi | 5 |
| 52 | Saluagaja | 3 | 228 | Dhanarasi | 5 |
| 53 | Basapatri | 1 | 229 | VL Dhan221 | 5 |
| 54 | Sankarachini | 3 | 230 | Vikash | 5 |
| 55 | Gajapati | 5 | 231 | Champeisiali | 3 |
| 56 | Rambha | 5 | 232 | Punjabniswarna | 5 |
| 57 | Samanta | 5 | 233 | Chakaakhi | 7 |
| 58 | Badami | 5 | 234 | PTB39 | 5 |
| 59 | Jajati | 5 | 235 | NLR33359 | 5 |
| 60 | CRM2203 | 5 | 236 | T23 | 5 |
| 61 | KJT2 | 5 | 237 | Kanchan | 5 |
| 62 | PTB38 | 5 | 238 | Kargi | 5 |
| 63 | Asinasita | 3 | 239 | Tulasi | 5 |
| 64 | Jagabalia | 3 | 240 | CSR13 | 5 |
| 65 | Kanatara | 3 | 241 | JM-10-31 | 5 |
| 66 | Lalgura | 3 | 242 | Subhadra | 5 |
| 67 | Jungajhata | 3 | 243 | Jogesh | 5 |
| 68 | Rangasiuli | 3 | 244 | Sarathi | 5 |
| 69 | Khandasagar | 3 | 245 | Sidhanta | 5 |
| 70 | Chanari | 3 | 246 | Mahalaxmi | 5 |
| 71 | Bsudha | 3 | 247 | Kharavela | 5 |
| 72 | Basudha | 5 | 248 | Hema | 5 |
| 73 | Kabir | 3 | 249 | Kalachampa | 5 |
| 74 | Tulasibasa | 3 | 250 | CR Dhan508 | 3 |
| 75 | Nalikalma | 3 | 251 | CR Dhan507 | 3 |
| 76 | Bhangar | 3 | 252 | CR Dhan506 | 3 |
| 77 | Malata | 5 | 253 | CR Dhan505 | 3 |
| 78 | Gobindabhog | 3 | 254 | Prachi | 5 |
| 79 | Abhaya | 1 | 255 | Bishnupriya | 3 |
| 80 | Agnisar | 9 | 256 | Tikimahsuri | 3 |
| 81 | Luna | 3 | 257 | Katak chara | 1 |
| 82 | Sebati | 9 | 258 | Kalajeera | 3 |
| 83 | Nadalghanta | 3 | 259 | Shasarang | 1 |
| 84 | Bhutmundi | 3 | 260 | LR 11 | 1 |
| 85 | Jata | 7 | 261 | Mirikarak | 3 |
| 86 | Sarubhajana | 3 | 262 | Pawnbuh | 3 |
| 87 | Tulasimali | 3 | 263 | Nemo | 3 |
| 88 | Abhiram | 7 | 264 | Pangkla | 3 |
| 89 | Pateni | 5 | 265 | Malliphulajhuli | 3 |
| 90 | Ahirman | 5 | 266 | Bharati | 3 |
| 91 | Phalguna | 3 | 267 | Mahsuri | 3 |
| 92 | Polkati | 3 | 268 | Makarkanda | 3 |
| 93 | Pokkali | 3 | 269 | IR32 | 1 |
| 94 | Khajurikandi | 5 | 270 | Sheetabhog | 1 |
| 95 | Sapri | 5 | 271 | Pankaj | 1 |
| 96 | Dhoiabankoi | 3 | 272 | Haribhog | 1 |
| 97 | Nalibaunsagaja | 7 | 273 | Kalamulia | 1 |
| 98 | Malabati | 9 | 274 | Gitanjali | 3 |
| 99 | Madhukar | 1 | 275 | Surendra | 3 |
| 100 | Nikipakhia | 7 | 276 | Swarna | 3 |
| 101 | Saraswati | 5 | 277 | Lalat | 3 |
| 102 | Jhilli | 5 | 278 | Sumit | 3 |
| 103 | Konark | 3 | 279 | IR36 | 3 |
| 104 | Hunder | 5 | 280 | CR683-195 | 3 |
| 105 | Chakau | 1 | 281 | Ambika | 1 |
| 106 | Labangalata | 5 | 282 | CR Dhan500 | 5 |
| 107 | Korkaili | 9 | 283 | OR1358-RGA-4 | 3 |
| 108 | Matiakhoja | 5 | 284 | Purnendu | 3 |
| 109 | Kusumkunda | 7 | 285 | Budhamanda | 3 |
| 110 | Mahanadi | 3 | 286 | CR595-25 | 3 |
| 111 | Kanchan | 3 | 287 | Padmakesari | 3 |
| 112 | Khajara | 3 | 288 | CR1005-1 | 3 |
| 113 | Gajapati | 3 | 289 | CR1006-1 | 3 |
| 114 | Mahamaya | 3 | 290 | Rambha | 3 |
| 115 | Sarathi | 3 | 291 | Ranidhan | 5 |
| 116 | RAU1326-94-2 | 3 | 292 | MTU1010 | 5 |
| 117 | RAU1326-29-5 | 3 | 293 | Mahipal | 5 |
| 118 | Reeta | 3 | 294 | Habira | 7 |
| 119 | OR1334-16 | 3 | 295 | Kanthakamal | 5 |
| 120 | Ruksal | 3 | 296 | Vijeta | 3 |
| 121 | Jagannath | 7 | 297 | Udaya | 3 |
| 122 | Manika | 3 | 298 | CR691-47 | 3 |
| 123 | Urbashi | 9 | 299 | CR662-2211 | 3 |
| 124 | Salivahan | 5 | 300 | Indravati | 3 |
| 125 | Maibetikela | 7 | 301 | Rambha | 3 |
| 126 | Kasalath | 7 | 302 | Abhaya | 3 |
| 127 | Prakash | 9 | 303 | Dubraj | 3 |
| 128 | Jyoti | 7 | 304 | Gajapati | 3 |
| 129 | Pyzum | 9 | 305 | Pusa 2-21 | 3 |
| 130 | IR64 | 7 | 306 | Srabani | 3 |
| 131 | IR26 | 7 | 307 | Triveni | 3 |
| 132 | IR42 | 7 | 308 | CR333-103 | 3 |
| 133 | Soyal Sali | 9 | 309 | OR1334-16 | 3 |
| 134 | CR992-5 | 9 | 310 | PY3 | 3 |
| 135 | Rayada-B3 | 7 | 311 | IR20 | 3 |
| 136 | CR385-2-4 | 9 | 312 | Komal-9 | 3 |
| 137 | CR256-M-42 | 9 | 313 | Himalay 741 | 3 |
| 138 | Sabita | 9 | 314 | Ravi | 3 |
| 139 | CR589-4-9 | 9 | 315 | PTB 5 | 3 |
| 140 | Heera | 7 | 316 | PTB 10 | 3 |
| 141 | Pathara | 7 | 317 | PTB 13 | 3 |
| 142 | Jajati | 7 | 318 | PTB 14 | 3 |
| 143 | Khitish | 7 | 319 | PTB 22 | 3 |
| 144 | Konark | 7 | 320 | PTB 23 | 3 |
| 145 | Sneha | 7 | 321 | Rajbandhu | 3 |
| 146 | Mahamaya | 7 | 322 | Bhubana | 3 |
| 147 | CR758-67 | 9 | 323 | Udayagiri | 3 |
| 148 | RR122-1 | 9 | 324 | Rudra | 3 |
| 149 | Daya | 7 | 325 | Khandagiri | 3 |
| 150 | Bharati | 7 | 326 | Rajeswari | 3 |
| 151 | Tulsi | 9 | 327 | Sankar | 3 |
| 152 | Mohan | 7 | 328 | Uphar | 3 |
| 153 | Rajeswari | 7 | 329 | Keshan | 3 |
| 154 | Krishna Hamsa | 7 | 330 | Bhaizata | 3 |
| 155 | Kranti | 7 | 331 | Mahanadi | 3 |
| 156 | Himalay | 7 | 332 | Tejaswini | 3 |
| 157 | IR50 | 7 | 333 | Manika | 3 |
| 158 | Parijat | 7 | 334 | Ghanteswari | 3 |
| 159 | Richaria | 9 | 335 | Daya | 3 |
| 160 | Sebati | 9 | 336 | Suphala | 3 |
| 161 | Mahalaxmi | 5 | 337 | Pratikshya | 5 |
| 162 | Harisankar | 1 | 338 | Parijata | 3 |
| 163 | Dimapur | 5 | 339 | Pratap | 3 |
| 164 | Sreebalaram | 1 | 340 | Ranisaheba | 5 |
| 165 | Dhanashree | 3 | 341 | Saluagaja | 3 |
| 166 | Khndiratnachudi | 3 | 342 | Tanmayee | 7 |
| 167 | Budidhan | 9 | 343 | Ashutosh | 5 |
| 168 | Karpuragundi | 7 | 344 | Hasanta | 5 |
| 169 | Dhoiamadhoi | 5 | 345 | Santepheap | 5 |
| 170 | Bagadachinamala | 7 | 346 | OR2327-23 | 7 |
| 171 | Kaniara | 5 | 347 | Ganjamgedi | 3 |
| 172 | Rasapanjari | 3 | 348 | Seulapana | 3 |
| 173 | Mayurachulia | 7 | 349 | Kadalipenda | 5 |
| 174 | Madhabi | 7 | 350 | Kakudimanji | 5 |
| 175 | Kalaheera | 3 | 351 | Bankoi | 7 |
| 176 | Nalijagannath | 3 | 352 | Laxmi | 9 |
