# Supplementary material for: Population genetic structure and association mapping for iron toxicity tolerance in rice
Source: PLoS One. 2021 Mar 1;16(3):e0246232. doi: 10.1371/journal.pone.0246232 (PMC7920388; doi:10.1371/journal.pone.0246232)
Supplement: S2 Table — (DOCX) [file pone.0246232.s003.docx]

**S2 Table**. List of molecular markers used for association analysis of Fe-toxicity tolerance in rice.

| Sl.No | Marker | Chr. No | Forward | Reverse |
| --- | --- | --- | --- | --- |
| 1 | RM488 | 1 | cagctagggttttgaggctg | tagcaacaaccagcgtatgc |
| 2 | RM243 | 1 | gatctgcagactgcagttgc | agctgcaacgatgttgtcc |
| 3 | RM490 | 1 | atctgcacactgcaaacacc | agcaagcagtgctttcagag |
| 4 | RM7102 | 12 | TTGAGAGCGTTTTTAGGATG | TCGGTTTACTTGGTTACTCG |
| 5 | RM17 | 12 | TGCCCTGTTATTTTCTTCTCT | GGTGATCCTTTCCCATTTCA |
| 6 | RM2416 | 4 | GTAGGTACATATGAATCCGA | TACAAATTAAAATAGGGGAA |
| 7 | RM307 | 4 | gtactaccgacctaccgttcac | ctgctatgcatgaactgctc |
| 8 | RM452 | 2 | ctgatcgagagcgttaaggg | gggatcaaaccacgtttctg |
| 9 | RM471 | 4 | acgcacaagcagatgatgag | gggagaagacgaatgtttgc |
| 10 | RM105 | 9 | gtcgtcgacccatcggagccac | tggtcgaggtggggatcgggtc |
| 11 | RM407 | 8 | gattgaggagacgagccatc | ctttttcagatctgcgctcc |
| 12 | RM3 | 6 | ACACTGTAGCGGCCACTG | CCTCCACTGCTCCACATCTT |
| 13 | RM31 | 5 | GATCACGATCCACTGGAGCT | AAGTCCATTACTCTCCTCCC |
| 14 | RM237 | 1 | caaatcccgactgctgtcc | tgggaagagagcactacagc |
| 15 | RM1278 | 9 | ATATAAAGGTGGCACGACAG | GCACTTGAACTCTAATTCTCC |
| 16 | RM590 | 10 | catctccgctctccatgc | ggagttggggtcttgttcg |
| 17 | RM260 | 12 | actccactatgacccagag | gaacaatcccttctacgatcg |
| 18 | RM234 | 7 | acagtatccaaggccctgg | cacgtgagacaaagacggag |
| 19 | RM248 | 7 | tccttgtgaaatctggtccc | gtagcctagcatggtgcatg |
| 20 | RM122 | 5 | gagtcgatgtaatgtcatcagtgc | gaaggaggtatcgctttgttggac |
| 21 | RM517 | 3 | ggcttactggcttcgatttg | cgtctcctttggttagtgcc |
| 22 | RM7003 | 12 | GGCAGACATACAGCTTATAGGC | TGCAAATGAACCCCTCTAGC |
| 23 | RM245 | 9 | atgccgccagtgaatagc | ctgagaatccaattatctgggg |
| 24 | RM3412 | 1 | AAAGCAGGTTTTCCTCCTCC | CCCATGTGCAATGTGTCTTC |
| 25 | RM6712 | 3 | GCGCATCATCACTTCATCAG | AGATGAGCCTATCAGCTGCC |
| 26 | RM432 | 7 | ttctgtctcacgctggattg | agctgcgtacgtgatgaatg |
| 27 | RM556 | 8 | actccaaacctcactgcacc | tagcacactgaacagctggc |
| 28 | RM269 | 10 | gaaagcgatcgaaccagc | gcaaatgcgcctcgtgtc |
| 29 | RM3331 | 12 | CCTCCTCCATGAGCTAATGC | AGGAGGAGCGGATTTCTCTC |
| 30 | RM202 | 11 | cagattggagatgaagtcctcc | ccagcaagcatgtcaatgta |
| 31 | RM168 | 3 | tgctgcttgcctgcttccttt | gaaacgaatcaatccacggc |
| 32 | RM5897 | 2 | GGCATCTTCCCCTCTCTCTC | CCAACCCAAACCAGTCTACC |
| 33 | RM5638 | 1 | GGCTTCCTCATCGCCATC | CTGAGCAGCATTCCAGTCTG |
| 34 | RM232 | 3 | ccggtatccttcgatattgc | ccgacttttcctcctgacg |
| 35 | RM8044 | 7 | AGTACTTGTCTCCTTAGCAG | CAATATTCACTCAACTCTCA |
| 36 | RM23 | 1 | acagtattccgtaggcacgg | gctccatgagggtggtagag |
| 37 | RM8007 | 7 | AATAGGATGGATCATGGATA | CATCTCATCAGGAACCTAAC |
| 38 | RM501 | 7 | gcccaattaatgtacaggcg | atatcgtttagccgtgctgc |
| 39 | RM574 | 5 | ggcgaattctttgcacttgg | acggtttggtagggtgtcac |
| 40 | RM585 | 6 | cagtcttgctccgtttgttg | ctgtgactgacttggtcatagg |
| 41 | RM440 | 5 | catgcaacaacgtcaccttc | atggttggtaggcaccaaag |
| 42 | RM594 | 1 | gccaccagtaaaagcaatac | ttgatctgctagtgagaccc |
| 43 | RM206 | 11 | cccatgcgtttaactattct | cgttccatcgatccgtatgg |
| 44 | RM152 | 8 | GAAACCACCACACCTCACCG | CCGTAGACCTTCTTGAAGTAG |
| 45 | RM205 | 9 | ctggttctgtatgggagcag | ctggcccttcacgtttcagtg |
| 46 | RM309 | 12 | gtagatcacgcacctttctgg | agaaggcctccggtgaag |
| 47 | RM7 | 3 | ttcgccatgaagtctctcg | cctcccatcatttcgttgtt |
| 48 | OsIRT1 | 3 | CGTCTTCTTCTTCTCCACCACGAC | GCAGCTGATGATCGAGTCTGACC |
| 49 | OsIRT2 | 7 | TCTTCCACCCTGAGCAGCTC | AACCTTGGAGACCAGTGCAG |
| 50 | Loc_Os01g49710 | 1 | CCTGGAGCACTACAAGGGAT | CGAGCAAGGCAGATAGATTG |
| 51 | Loc_Os01g49720 | 1 | CGTCGTCCTGGAGTACATC | GACCTGAACAGCACTTTCC |
