# Supplementary material for: Population genetic structure and association mapping for iron toxicity tolerance in rice
Source: PLoS One. 2021 Mar 1;16(3):e0246232. doi: 10.1371/journal.pone.0246232 (PMC7920388; doi:10.1371/journal.pone.0246232)
Supplement: S3 Table — (DOCX) [file pone.0246232.s004.docx]

**S3 Table**. Molecular diversity parameters estimates from 119 genotypes using 51 molecular markers

| **Sl.No.** | **Marker** | **Allele**  **size (bp)** | **Major**  **allele**  **frquency** | **No. of allele** | **Gene**  **Diversity** | **Heterozygosity** | **PIC** | **Inbreeding coefficient (f)** |
| --- | --- | --- | --- | --- | --- | --- | --- | --- |
| 1 | RM488 | 150-200 | 0.4804 | 4 | 0.5618 | 0.1765 | 0.4643 | 0.6885 |
| 2 | RM243 | 90-120 | 0.6623 | 3 | 0.4731 | 0.0789 | 0.3953 | 0.8345 |
| 3 | RM490 | 100-120 | 0.7783 | 2 | 0.3451 | 0.0087 | 0.2856 | 0.9750 |
| 4 | RM7102 | 170-200 | 0.9344 | 2 | 0.1225 | 0.0328 | 0.1150 | 0.7363 |
| 5 | RM17 | 160-200 | 0.6681 | 2 | 0.4435 | 0.0431 | 0.3451 | 0.9036 |
| 6 | RM2416 | 250-270 | 0.5942 | 2 | 0.4823 | 0.0000 | 0.3660 | 1.0000 |
| 7 | RM307 | 120-200 | 0.8378 | 3 | 0.2780 | 0.0631 | 0.2499 | 0.7750 |
| 8 | RM452 | 240-265 | 0.4639 | 3 | 0.6239 | 0.0206 | 0.5466 | 0.9673 |
| 9 | RM471 | 110-135 | 0.7383 | 4 | 0.4213 | 0.0467 | 0.3842 | 0.8901 |
| 10 | RM105 | 100-140 | 0.6471 | 3 | 0.5190 | 0.3235 | 0.4649 | 0.3808 |
| 11 | RM407 | 160-190 | 0.6934 | 3 | 0.4280 | 0.0566 | 0.3404 | 0.8689 |
| 12 | RM3 | 110-150 | 0.7699 | 4 | 0.3806 | 0.2301 | 0.3487 | 0.3992 |
| 13 | RM31 | 90-110 | 0.5510 | 3 | 0.5923 | 0.1020 | 0.5243 | 0.8293 |
| 14 | RM237 | 130-150 | 0.6346 | 3 | 0.5259 | 0.0192 | 0.4665 | 0.9638 |
| 15 | RM1278 | 135-150 | 0.5625 | 3 | 0.5873 | 0.0673 | 0.5219 | 0.8864 |
| 16 | RM590 | 130-150 | 0.5088 | 3 | 0.6111 | 0.0265 | 0.5375 | 0.9569 |
| 17 | RM260 | 140-190 | 0.7500 | 3 | 0.4053 | 0.4483 | 0.3687 | -0.1017 |
| 18 | RM234 | 130-150 | 0.8187 | 2 | 0.2969 | 0.0330 | 0.2528 | 0.8901 |
| 19 | RM248 | 70-100 | 0.5270 | 3 | 0.5592 | 0.2162 | 0.4669 | 0.6162 |
| 20 | RM122 | 230-260 | 0.4451 | 4 | 0.6013 | 0.4505 | 0.5172 | 0.2559 |
| 21 | RM517 | 250-280 | 0.7363 | 3 | 0.4096 | 0.0549 | 0.3569 | 0.8672 |
| 22 | RM7003 | 90-110 | 0.6556 | 3 | 0.5109 | 0.1778 | 0.4581 | 0.6552 |
| 23 | RM245 | 150-155 | 0.9024 | 3 | 0.1797 | 0.0000 | 0.1700 | 1.0000 |
| 24 | RM3412 | 200-260 | 0.3679 | 4 | 0.6922 | 0.1604 | 0.6314 | 0.7703 |
| 25 | RM6712 | 110-140 | 0.3621 | 4 | 0.6680 | 0.1494 | 0.5955 | 0.7786 |
| 26 | RM432 | 180-190 | 0.5733 | 2 | 0.4893 | 0.0086 | 0.3696 | 0.9825 |
| 27 | RM556 | 180-180 | 1.0000 | 1 | 0.0000 | 0.0000 | 0.0000 | NaN |
| 28 | RM269 | 180-310 | 0.3640 | 4 | 0.7149 | 0.8596 | 0.6626 | -0.1983 |
| 29 | RM3331 | 160-190 | 0.6935 | 3 | 0.4715 | 0.1613 | 0.4248 | 0.6625 |
| 30 | RM202 | 160-200 | 0.4842 | 3 | 0.6232 | 0.3158 | 0.5487 | 0.4972 |
| 31 | RM168 | 90-125 | 0.8333 | 3 | 0.2810 | 0.0417 | 0.2470 | 0.8546 |
| 32 | RM5897 | 130-160 | 0.5963 | 4 | 0.5678 | 0.0000 | 0.5111 | 1.0000 |
| 33 | RM5638 | 205-250 | 0.4524 | 4 | 0.6240 | 0.1905 | 0.5462 | 0.6972 |
| 34 | RM232 | 150-180 | 0.7526 | 3 | 0.3858 | 0.0412 | 0.3315 | 0.8942 |
| 35 | RM8044 | 250-300 | 0.4381 | 3 | 0.6321 | 0.1524 | 0.5547 | 0.7609 |
| 36 | RM23 | 140-150 | 0.5156 | 2 | 0.4995 | 0.0104 | 0.3748 | 0.9794 |
| 37 | RM8007 | 140-160 | 0.5074 | 2 | 0.4999 | 0.1029 | 0.3749 | 0.7968 |
| 38 | RM501 | 140-160 | 0.5053 | 2 | 0.4999 | 0.4787 | 0.3750 | 0.0478 |
| 39 | RM574 | 150-170 | 0.9247 | 2 | 0.1393 | 0.1233 | 0.1296 | 0.1220 |
| 40 | RM585 | 130-150 | 0.5625 | 3 | 0.5844 | 0.0313 | 0.5174 | 0.9471 |
| 41 | RM440 | 150-200 | 0.8347 | 3 | 0.2891 | 0.2119 | 0.2694 | 0.2711 |
| 42 | RM594 | 300-320 | 0.7198 | 2 | 0.4034 | 0.1121 | 0.3220 | 0.7242 |
| 43 | RM206 | 140-170 | 0.2889 | 4 | 0.7449 | 0.1556 | 0.6972 | 0.7932 |
| 44 | RM152 | 140-160 | 0.5146 | 3 | 0.5563 | 0.7864 | 0.4600 | -0.4095 |
| 45 | RM205 | 100-170 | 0.9237 | 3 | 0.1421 | 0.0169 | 0.1341 | 0.8816 |
| 46 | RM309 | 180-210 | 0.7632 | 3 | 0.3871 | 0.1140 | 0.3512 | 0.7076 |
| 47 | RM7 | 200-250 | 0.5164 | 2 | 0.4995 | 0.9672 | 0.3747 | -0.9355 |
| 48 | OsIRT1 | 350-380 | 0.8933 | 2 | 0.1907 | 0.2135 | 0.1725 | -0.1139 |
| 49 | OsIRT2 | 230-240 | 0.9677 | 2 | 0.0624 | 0.0000 | 0.0605 | 1.0000 |
| 50 | Loc_Os01g49710 | 90-90 | 1.0000 | 1 | 0.0000 | 0.0000 | 0.0000 | NaN |
| 51 | Loc_Os01g49720 | 220-220 | 1.0000 | 1 | 0.0000 | 0.0000 | 0.0000 | NaN |
|  | Mean |  | 0.6611 | 2.80 | 0.4315 | 0.1585 | 0.3722 | 0.6360 |
