# Supplementary material for: Population genetic structure and association mapping for iron toxicity tolerance in rice
Source: PLoS One. 2021 Mar 1;16(3):e0246232. doi: 10.1371/journal.pone.0246232 (PMC7920388; doi:10.1371/journal.pone.0246232)
Supplement: S4 Table — (DOCX) [file pone.0246232.s005.docx]

**S4 Table.** The inferred ancestry value and population structure of the members in a panel containing119 genotypes with their response to Fe-toxicity tolerance

| Sl.  No. | Genotype Name | Inferred ancestry at K=3 | | | Structure  group | Genotype response to the Fe-toxicity stress |
| --- | --- | --- | --- | --- | --- | --- |
|  |  | Q1 | Q2 | Q3 |  |  |
| 1 | Sankaribako | 0.921 | 0.009 | 0.06 | SP1 | MR |
| 2 | Kalakrushna | 0.979 | 0.011 | 0.01 | SP1 | MS |
| 3 | Assamchudi | 0.979 | 0.01 | 0.011 | SP1 | MR |
| 8 | GeleiA | 0.953 | 0.013 | 0.034 | SP1 | MS |
| 12 | Jubaraj | 0.905 | 0.026 | 0.07 | SP1 | R |
| 23 | Ratanmali | 0.891 | 0.075 | 0.034 | SP1 | MR |
| 25 | Umarcudi | 0.939 | 0.028 | 0.033 | SP1 | R |
| 27 | Anu | 0.902 | 0.04 | 0.058 | SP1 | MR |
| 29 | Ramakrushanabilash | 0.912 | 0.065 | 0.02 | SP1 | MR |
| 30 | GeleiB | 0.891 | 0.054 | 0.055 | SP1 | R |
| 33 | Juiphula | 0.906 | 0.08 | 0.015 | SP1 | R |
| 34 | Karpurakranti | 0.875 | 0.11 | 0.01 | SP1 | MS |
| 37 | Pipalbasa | 0.88 | 0.019 | 0.101 | SP1 | MR |
| 38 | Jaiphula | 0.905 | 0.029 | 0.066 | SP1 | MR |
| 42 | Khandasagar | 0.939 | 0.038 | 0.02 | SP1 | MS |
| 44 | Kusuma | 0.804 | 0.022 | 0.174 | SP1 | MR |
| 45 | Kendrajhali | 0.839 | 0.021 | 0.14 | SP1 | R |
| 48 | Basapatri | 0.831 | 0.016 | 0.153 | SP1 | R |
| 49 | Kalaheera | 0.884 | 0.071 | 0.045 | SP1 | MR |
| 50 | Budidhan | 0.954 | 0.014 | 0.033 | SP1 | S |
| 51 | Karpuragundi | 0.977 | 0.015 | 0.008 | SP1 | MS |
| 53 | Bagadachinamala | 0.972 | 0.014 | 0.01 | SP1 | MS |
| 76 | Luna | 0.821 | 0.125 | 0.054 | SP1 | MR |
| 5 | Champa | 0.785 | 0.05 | 0.165 | SP1 -Admix | S |
| 26 | Nilarpati | 0.78 | 0.039 | 0.181 | SP1 -Admix | MR |
| 31 | Sunapani | 0.675 | 0.169 | 0.156 | SP1 -Admix | MS |
| 35 | Ranisaheba | 0.535 | 0.025 | 0.44 | SP1 -Admix | MR |
| 39 | Mayurkantha | 0.562 | 0.015 | 0.42 | SP1 -Admix | MS |
| 77 | Sebati | 0.746 | 0.221 | 0.033 | SP1 -Admix | S |
| 82 | Tulasimali | 0.62 | 0.321 | 0.059 | SP1 -Admix | R |
| 83 | Abhiram | 0.61 | 0.199 | 0.191 | SP1 -Admix | MS |
| 36 | Mahipal | 0.465 | 0.082 | 0.453 | SP1 –Admix | MR |
| 80 | Jata | 0.023 | 0.962 | 0.015 | SP2 | MS |
| 87 | Makarkanda | 0.017 | 0.963 | 0.02 | SP2 | R |
| 101 | Labangalata | 0.04 | 0.939 | 0.02 | SP2 | MR |
| 106 | Kanchan | 0.071 | 0.809 | 0.12 | SP2 | MR |
| 108 | Rambha | 0.027 | 0.946 | 0.027 | SP2 | MR |
| 109 | Mahalaxmi | 0.065 | 0.92 | 0.014 | SP2 | MS |
| 110 | Harisankar | 0.009 | 0.982 | 0.008 | SP2 | R |
| 112 | Sreebalaram | 0.117 | 0.826 | 0.05 | SP2 | MR |
| 113 | Dhanashree | 0.02 | 0.919 | 0.061 | SP2 | MR |
| 114 | Khndiratnachudi | 0.009 | 0.969 | 0.02 | SP2 | MR |
| 115 | Ruksal | 0.091 | 0.877 | 0.032 | SP2 | MR |
| 116 | Jagannath | 0.017 | 0.949 | 0.034 | SP2 | MS |
| 117 | Manika | 0.009 | 0.981 | 0.01 | SP2 | MR |
| 118 | Urbashi | 0.034 | 0.955 | 0.011 | SP2 | S |
| 119 | Salivahan | 0.011 | 0.981 | 0.008 | SP2 | MR |
| 20 | Chudi | 0.057 | 0.488 | 0.455 | SP2 -Admix | MS |
| 68 | Kabir | 0.221 | 0.513 | 0.266 | SP2 -Admix | R |
| 70 | Nalikalma | 0.083 | 0.574 | 0.343 | SP2 -Admix | R |
| 85 | Ahirman | 0.096 | 0.691 | 0.213 | SP2 -Admix | MR |
| 89 | Khajurikandi | 0.165 | 0.783 | 0.05 | SP2 -Admix | MR |
| 100 | Haribhog | 0.016 | 0.714 | 0.27 | SP2 -Admix | R |
| 105 | Padmakesari | 0.041 | 0.591 | 0.36 | SP2 -Admix | R |
| 111 | Dimapur | 0.21 | 0.656 | 0.134 | SP2 -Admix | MR |
| 4 | Nini | 0.075 | 0.033 | 0.892 | SP3 | S |
| 6 | Mugei | 0.061 | 0.037 | 0.902 | SP3 | MR |
| 7 | Latamahu | 0.147 | 0.024 | 0.829 | SP3 | MS |
| 10 | Veleri | 0.016 | 0.016 | 0.969 | SP3 | MR |
| 13 | Dhabalabhuta | 0.043 | 0.016 | 0.94 | SP3 | MR |
| 15 | Dhinkisiali | 0.018 | 0.01 | 0.97 | SP3 | MS |
| 16 | Sagiri | 0.136 | 0.012 | 0.852 | SP3 | MR |
| 17 | Bayabhanda | 0.125 | 0.023 | 0.852 | SP3 | R |
| 19 | Hatipanjara | 0.095 | 0.054 | 0.85 | SP3 | MS |
| 22 | Kakiri | 0.037 | 0.042 | 0.921 | SP3 | MR |
| 24 | Dhusura | 0.048 | 0.025 | 0.926 | SP3 | R |
| 28 | Madia | 0.053 | 0.011 | 0.936 | SP3 | MS |
| 32 | Jabaphula | 0.238 | 0.012 | 0.75 | SP3 | R |
| 40 | Champeisiali | 0.074 | 0.022 | 0.9 | SP3 | R |
| 41 | Nalijagannath | 0.079 | 0.079 | 0.84 | SP3 | MS |
| 52 | Dhoiamadhoi | 0.047 | 0.031 | 0.92 | SP3 | MR |
| 56 | Mayurachulia | 0.017 | 0.043 | 0.93 | SP3 | MS |
| 57 | Madhabi | 0.013 | 0.023 | 0.964 | SP3 | MS |
| 58 | Rangasiuli | 0.112 | 0.067 | 0.82 | SP3 | MR |
| 59 | Saluagaja | 0.009 | 0.017 | 0.974 | SP3 | R |
| 60 | Bishnupriya | 0.01 | 0.011 | 0.98 | SP3 | R |
| 63 | Asinasita | 0.031 | 0.017 | 0.953 | SP3 | R |
| 64 | Sankarachini | 0.018 | 0.032 | 0.95 | SP3 | MR |
| 67 | Basudha | 0.028 | 0.079 | 0.893 | SP3 | MR |
| 71 | Bhangar | 0.012 | 0.021 | 0.968 | SP3 | MR |
| 75 | Agnisar | 0.051 | 0.02 | 0.929 | SP3 | S |
| 86 | Malliphulajhuli | 0.015 | 0.168 | 0.81 | SP3 | MR |
| 88 | Bharati | 0.013 | 0.047 | 0.94 | SP3 | MR |
| 93 | Malabati | 0.024 | 0.058 | 0.918 | SP3 | S |
| 94 | Kalamulia | 0.048 | 0.017 | 0.935 | SP3 | R |
| 95 | Nikipakhia | 0.015 | 0.184 | 0.801 | SP3 | MS |
| 96 | Saraswati | 0.01 | 0.045 | 0.945 | SP3 | MR |
| 98 | Budhamanda | 0.012 | 0.022 | 0.966 | SP3 | MR |
| 99 | Hunder | 0.019 | 0.052 | 0.928 | SP3 | MR |
| 102 | Korkaili | 0.076 | 0.013 | 0.911 | SP3 | S |
| 104 | Kusumkunda | 0.031 | 0.085 | 0.883 | SP3 | MS |
| 107 | Khajara | 0.026 | 0.056 | 0.918 | SP3 | R |
| 9 | Kalamara | 0.415 | 0.106 | 0.479 | SP3 - Admix | MR |
| 11 | Gurumukhi | 0.266 | 0.021 | 0.713 | SP3 -Admix | R |
| 14 | Bangali | 0.078 | 0.263 | 0.659 | SP3 -Admix | S |
| 18 | Banda | 0.187 | 0.289 | 0.524 | SP3 -Admix | R |
| 21 | Jalpaya | 0.278 | 0.052 | 0.671 | SP3 -Admix | R |
| 43 | Punjabniswarna | 0.431 | 0.031 | 0.53 | SP3 -Admix | MR |
| 46 | Biridibankoi | 0.379 | 0.151 | 0.46 | SP3 -Admix | MR |
| 47 | Jagabalia | 0.084 | 0.133 | 0.783 | SP3 -Admix | MR |
| 54 | Kaniara | 0.102 | 0.151 | 0.747 | SP3 -Admix | MR |
| 55 | Rasapanjari | 0.222 | 0.044 | 0.73 | SP3 -Admix | R |
| 61 | Tikimahsuri | 0.05 | 0.238 | 0.71 | SP3 -Admix | R |
| 62 | Jungajhata | 0.026 | 0.282 | 0.692 | SP3 -Admix | R |
| 65 | Kalajeera | 0.105 | 0.136 | 0.759 | SP3 -Admix | MR |
| 66 | Bsudha | 0.037 | 0.381 | 0.582 | SP3 -Admix | MR |
| 69 | Tulasibasa | 0.198 | 0.154 | 0.648 | SP3 -Admix | MR |
| 72 | Malata | 0.259 | 0.257 | 0.484 | SP3 -Admix | MR |
| 73 | Gobindabhog | 0.262 | 0.083 | 0.65 | SP3 -Admix | MR |
| 74 | Latachaunri | 0.355 | 0.19 | 0.45 | SP3 -Admix | R |
| 78 | Nadalghanta | 0.46 | 0.048 | 0.49 | SP3 -Admix | MR |
| 79 | Bhutmundi | 0.091 | 0.299 | 0.61 | SP3 -Admix | R |
| 81 | Sarubhajana | 0.31 | 0.111 | 0.57 | SP3 -Admix | R |
| 84 | Pateni | 0.031 | 0.449 | 0.52 | SP3 -Admix | MR |
| 90 | Sapri | 0.306 | 0.114 | 0.58 | SP3 -Admix | MR |
| 91 | Dhoiabankoi | 0.302 | 0.024 | 0.67 | SP3 -Admix | R |
| 92 | Nalibaunsagaja | 0.016 | 0.266 | 0.71 | SP3 -Admix | MS |
| 97 | Jhilli | 0.014 | 0.209 | 0.776 | SP3 -Admix | MR |
| 103 | Matiakhoja | 0.035 | 0.212 | 0.754 | SP3 -Admix | MR |
